# Supplementary material for: In Vitro and In Silico Studies of Human Tyrosyl-DNA Phosphodiesterase 1 (Tdp1) Inhibition by Stereoisomeric Forms of Lipophilic Nucleosides: The Role of Carbohydrate Stereochemistry in Ligand-Enzyme Interactions
Source: Molecules. 2022 Apr 9;27(8):2433. doi: 10.3390/molecules27082433 (PMC9024977; doi:10.3390/molecules27082433)
Supplement: Supplementary file 1 [file molecules-27-02433-s001.zip › Supplementary.pdf]

In vitro and *in silico* studies of human DNA-tyrosyl phosphodiesterase 1 (Tdp1) inhibition by stereoisomeric forms of lipophilic nucleosides: The role of carbohydrate stereochemistry in ligand-enzyme interactions

Nadezhda S. Dyrkheeva [1], Irina A. Chernyshova [1], Georgy A. Ivanov [2], Yuri B. Porozov [2,3,4], Anastasia A. Zenchenko [2], Vladimir E. Oslovsky [2], Alexandra L. Zakharenko [1,5], Darina I. Nasyrova [6], Galina N. Likhatskaya [7], Sergey N. Mikhailov [2,†], Olga I. Lavrik [1,5,*] and Mikhail S. Drenichev [2,*]

[1] Institute of Chemical Biology and Fundamental Medicine, Siberian Branch of the Russian Academy of Sciences, 8 Lavrentiev Ave., Novosibirsk, 630090, Russian Federation
[2] Engelhardt Institute of Molecular Biology, Russian Academy of Sciences, 32 Vavilova Str., 119991, Moscow, Russian Federation
[3] World-Class Research Center "Digital Biodesign and Personalized Healthcare", I.M. Sechenov First Moscow State Medical University, 8/2, Trubetskaya St., Moscow 119991, Russian Federation
[4] Department of Computational Biology, Sirius University of Science and Technology, Olympic Ave. 1, 354340 Sochi, Russian Federation
[5] Department of Physical and Chemical Biology and Biotechnology, Altai State University, 656049 Barnaul, Russian Federation
[6] National Research University Higher School of Economics, 11-10, Pokrovsky Boulevard, 101000 Moscow, Russian Federation
[7] Laboratory of Bioassays and Mechanism of Action of Biologically Active Compounds, G.B. Elyakov Pacific Institute of Bioorganic Chemistry Far Eastern Branch of Russian Academy of Sciences, Prospekt 100 let Vladivostoky, 159, Vladivostok, 690022, Russian Federation
[†] Deceased
* Correspondence: mdrenichev@mail.ru (Drenichev M.S.); o_lavrik@ngs.ru (Lavrik O.I.), Tel.: +7-499-135-97-33 (Drenichev M.S.), +7-383-363-51-95 (Lavrik O.I.).

Chemistry. There are two main methods for obtaining nucleoside analogues. The first method is based on the modification of natural nucleosides [1]. The second method is based on condensation of heterocyclic bases with activated nucleoside derivatives with a formation of *N*-glycosidic bond [2]. To date, convenient and efficient methods for the synthesis of ribonucleosides have been developed, based on trimethylsilyl derivatives of heterocyclic bases and fully acylated ribofuranose in the presence of Lewis acids [3-4].

Glycosyl-donors **1 DL**-**2 DL** were prepared starting from commercially available carbohydrates according to the procedure [5]. *O*-Benzoylated derivatives of *D*-ribothymidine **4D**, 6-methyl-β-*D*-ribofuranosyluracil **4'D**, *L*-uridine **3L**, *L*-ribothymidine **4L** were synthesized according to the procedure, elaborated by Vorbruggen and-co-workers [3-4], starting from corresponding heterocyclic bases and 1-*O*-acetyl-2,3,5-tri-*O*-benzoyl-D(L) pentafuranoses in the presence of hexamethyldisilazane (HMDS) or *N*,*O*-*bis*-trimethylsilylacetamide (BSA) and trimethylsilyl triflate (TMSOT*f*) or $SnCl_4$. *O*-Benzoyl stereoisomers of 5'*C*-methyl-nucleosides **5R-7R** and **5S-7S** derivative were synthesized according to the procedure, elaborated by Reist and collegues [6], starting from corresponding heterocyclic bases and 1-*O*-acetyl-2,3,5-tri-*O*-benzoyl-D(L) pentafuranoses in the presence of hexamethyldisilazane (HMDS) or *N*,*O*-*bis*-trimethylsilylacetamide

(BSA) and trimethylsilyl triflate (TMSOT*f*) or $SnCl_4$. 2′,3′,5′-Tri-*O*-benzoyluridine **3D** was synthesized starting from uridine by its direct *O*-benzoylation with benzoyl cyanide in the presence of triethylamine at ambient temperature in 70% yield according to the procedure elaborated by Prasad and co-workers (**Scheme 1**).

The stereoselectivity of the reaction is determined by the 2-*O*-benzoyl group involved in the formation of the benzoyloxonium ion, thereby directing the reaction to the formation of β-nucleosides, in which the heterocyclic base is in the *trans* position with respect to the 2-*O*-benzoyl group. The configuration of *N*-glycosidic bond in pyrimidine pentafuranosyl nucleosides is confirmed by the presence in ^{1}H-NMR spectra of H-1′ signal as a doublet with $J_{1',2'} \sim 4\ Hz$ at 6.0-6.3 ppm. Pyrine analogues are characterized by H-1′ signal as a singlet. ^{1}H-NMR spectra of *L*-isomers did not have notable differences with corresponding spectra of *D*-isomers. The presence of methyl group in 5′*C*-Methyl derivatives was evidenced by the presence of characteristic proton resonance signal as doublet in high-field region in ^{1}H-NMR (δ ~ 1.4 ppm) and characteristic carbon resonance signal at δ ~ 16 ppm in ^{13}C-NMR (see the NMR spectra below).

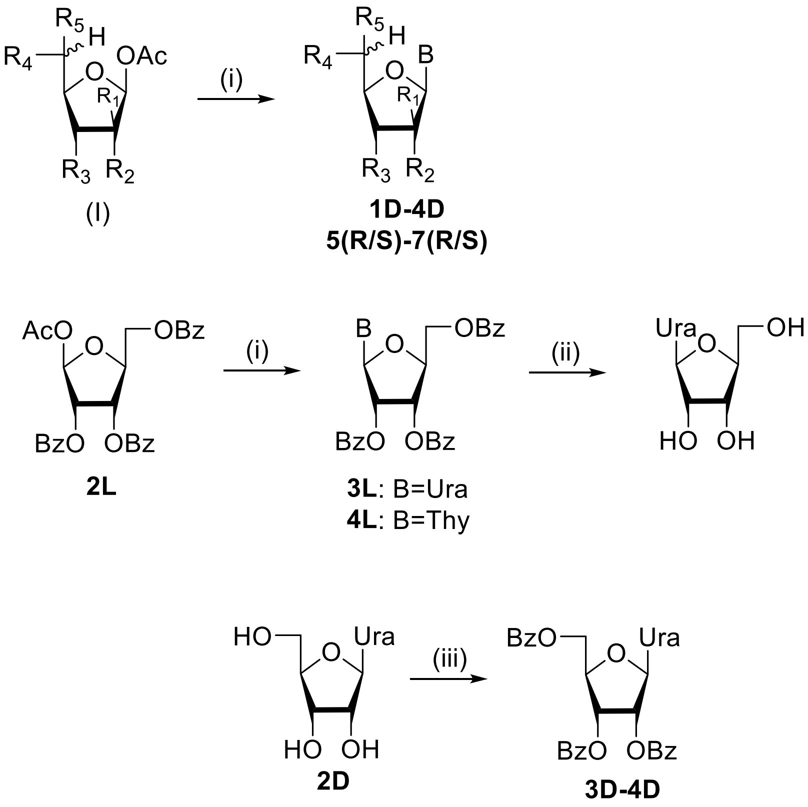

Scheme 1. Synthesis of lipophilic nucleoside derivatives starting from D- and L-pentafuranoses.
Reagents and conditions: (i) Ura or Thy or Cyt or *N*^{6}-BzAde, BSA, TMSOTf, DCE, Δ 52-60%; (ii) B=Ura: 4M NH_3/MeOH, 3 days, 90%; (iii) BzCN/Et_3N, dioxane, 40 min, r.t., 70%.

Example 1. Synthesis of 1-[2,3,5-Tri-*O*-benzoyl-β-D-ribofuranose-1-yl]uracil (3D) by *O*-benzoylation of uridine.

To a solution of uridine (1g, 4.7 mmol, 1 eq) in dry dioxane (40 mL), benzoyl cyanide (BzCN) (2.03 g, 15.51 mmol) and triethylamine (2.2 ml, 15.51 mmol) were added in one portionand the reaction mixture was stirred at ambient temperature for 40 min to full dissolving of BzCN. The reaction mixture was then treated with 15 mL of MeOH. The resulting solution was left to stay for 30 min at ambient temperature and then evaporated in vacuum. The residue was co-evaporated with CH_2Cl_2(10 mL). The product was crystallized from CH_2Cl_2 (3 mL). The precipitate was filtered, washed with mixture CH_2Cl_2 (3×2 mL) and dried in vacuum desiccator over P_2O_5 to yield1.84 g (70%) of white crystals. M.p. 153°C. R_f = 0.45 (CH_2Cl_2/EtOH- 99/1, v/v).

R_f = 0.45 (CH_2Cl_2/EtOH- 99/1, v/v). ^{1}H-NMR (300 MHz, DMSO-d_6): 11.49 (d, ^4J = 2.1 Hz, 1H, NH^3), 8.04 (dd, 2H, ^3J = 8.5 Hz, ^4J = 1.4 Hz, *o*-Bz), 7.93-7.85 (m, 4H, *o*-Bz), 7.83 (d, 1H, $^3J_{6\text{-}5}$ = 8.1 Hz, H-6 Ura), 7.68 – 7.60 (m, 3H, *p*-Bz), 7.52 (dd, 2H, ^3J = 8.5 Hz, ^3J = 6.9 Hz, *m*-Bz), 7.49-7.41 (m, 4H, *m*-Bz), 6.16 (d, $J_{1',2'}$ = 3.6 Hz, 1H, H-1′), 5.97-5.88 (m, 2H, H-2′, H-3′), 5.67 (dd, $^2J_{5\text{-}6}$= 8.1 Hz, ^4J = 2.1 Hz, 1H, H-5 Ura), 4.74 (ddd, $J_{4',3'}$ = 6.2 Hz, $J_{4',5'a}$ = 3.6 Hz, $J_{4',5'b}$ = 5.5 Hz, 1H, H-4′), 4.72 (dd, 1H, $J_{5'a,4'}$ = 3.6 Hz, $J_{5'a,5'b}$ = - 11.8 Hz, H-5′b), 4.64 (1H, $J_{5'b,4'}$ = 5.5 Hz, $J_{5'b,5'a}$ = -11.8 Hz, H-5′b). ^{13}C-NMR (75 MHz, DMSO-d_6): 165.44 (C=O), 164.60 (C=O), 164.57 (C=O), 163.04 (C-4), 150.27 (C-2), 142.20 (C-6), 133.88, 133.78, 133.48, 129.27 (Bz), 129.18 (Bz), 128.69 (Bz), 128.53 (Bz), 128.43 (Bz), 102.24 (C-5), 89.55 (C-1′), 78.74 (C-4′), 73.16 (C-2′), 70.49 (C-3′), 63.62 (C-5′).

Example 2. Synthesis of 1-[2,3,5-Tri-*O*-benzoyl-β-L-ribofuranose-1-yl]uracil (3L) by *N*-glycosylation of uracil.

A mixture of uracil (54 mg, 0.48 mmol) and *N*,*O*-bis(trimethylsilyl)acetamide (BSA, 0.176 mL, 0.72 mmol) was refluxed for 40 min until the formation of transparent solution. 1-*O*-acetyl-2,3,5-tri-*O*-benzoyl-β-L-ribofuranose (120 mg, 0.24 mmol) and triflate (TMSOTf, 0.65 mL, 0.35 mmol) were successively added to the solution. The resulting mixture was refluxed at 60°C for 3 hrs. and was then cooled to ambient temperature and neutralized with 10% aqueous sodium bicarbonate (20 mL). The product was extracted with methylene chloride (2×20 mL), the combined organic layers were separated, washed with distilled water (2×20 mL), dried over anhydrous sodium sulfate, filtered by gravity filtration and evaporated in vacuum to dryness. The residue was applied on chromatographic column with silica-gel (12 mL) for purification in system methylene chloride/ethanol - 99.4/0.6 (*v*/*v*). The fractions, containing the product, were collected and evaporated in vacuum to dryness. The residue was dried on a vacuum oil pump for 1.5 h. Yield 66 mg (49%) as white foam. R_f = 0.52 (CH_2Cl_2/EtOH - 99.4/0.6, *v*/*v*). ^{1}H-NMR is identical to **3D**.

1-[2,3,5-Tri-*O*-benzoyl-β-D-ribofuranose-1-yl]thymine (4D).

The procedure is analogous to the preparation of **3L** starting from thymine (75 mg, 0.6 mmol) and 1-*O*-acetyl-2,3,5-tri-*O*-benzoyl-β-L-ribofuranose (200 mg, 0.4 mmol). Yield 278 mg (82%) as foam. R_f = 0.55 (CH_2Cl_2/EtOH = 99/1, v/v). ^{1}H-NMR (300 MHz, DMSO-d_6): 11.46 (s, ^{3}NH), 8.03 (dd, 2H, ^3J = 8.5 Hz, ^4J = 1.4 Hz, *o*-Bz), 7.91 (dd, 2H, ^3J = 8.5 Hz, ^4J = 1.4 Hz, *o*-Bz), 7.87 (dd, 1H, ^3J = 8.5 Hz, ^4J = 1.4 Hz, *o*-Bz), 7.71-7.60 (m, 4H, *p*-Bz+H-6 Thy), 7.52 (dd, 2H, ^3J = 7.6 Hz, ^3J = 8.5 Hz, *m*-Bz), 7.49-7.40 (m, 4H, *m*-Bz), 6.20 (d, $J_{1',2'}$ = 4.2 Hz, 1H, H-1′), 5.96-5.87 (m, 2H, H-2′, H-3′), 4.78-4.73 (m, 1H, H-4′, overlapping withH-5′a), 4.73 (dd, 1H, $J_{5'a,4'}$ = 3.5 Hz, $J_{5'a,5'b}$ = - 12.5 Hz, H-5′a), 4.63 (1H, $J_{5'b,4'}$ = 5.8 Hz, $J_{5'b,5'a}$ = -12.5 Hz, H-5′b), 1.68 (s, 3H, Me). ^{13}C-NMR (75 MHz, DMSO-d_6): 165.44 (C=O), 164.61 (C=O), 164.55 (C=O), 163.62 (C-4), 150.34 (C-2), 137.03 (C-6), 133.89, 133.79, 133.54, 129.28, 129.18, 129.06, 128.75, 128.68, 128.55, 128.35 (Bz), 110.08 (C-5), 88.38 (C-1′), 78.73 (C-4′), 73.02 (C-2′), 70.56 (C-3′), 63.64 (C-5′), 11.83 (Me).

1-[2,3,5-Tri-*O*-benzoyl-β-D-ribofuranose-1-yl]thymine (4L).

The procedure is analogous to the preparation of **3L** starting from thymine (75 mg, 0.6 mmol) and 1-*O*-acetyl-2,3,5-tri-*O*-benzoyl-β-L-ribofuranose (200 mg, 0.4 mmol). Yield 14 mg (50%) as foam. R_f = 0.12 (CH_2Cl_2/EtOH = 99.4/0.6, v/v). ^{1}H-NMR is identical to **4D**.

Literature

1. M.S. Drenichev, V.E. Oslovsky, S.N. Mikhailov. Cytokinin Nucleosides - Natural compounds with a unique spectrum of biological activities. *Curr. Top. Med. Chem.*, **2016**, 16, 2562-2576, DOI: 10.2174/1568026616666160414123717.

2. Mikhailov, S.N.; Efimtseva, E.V.; Rodionov, A.A.; Bobkov, G.V.; Kulikova, I.V.; Herdewijn, P. Synthesis of 2-O-β-D-ribofuranosylnucleosides. *Curr. Prot. Nucl. Acid Chem*. **2006**, 27, 1.14.1–1.14.18, doi:10.1002/0471142700.nc0114s27.

3. H.Vorbrüggen, C. Ruh-Pohlenz. Handbook of nucleoside synthesis (Vol. 60). John Wiley & Sons, 2001.

4. Vorbrüggen, H. Adventures in silicon-organic chemistry. *Acc. Chem. Res*. **1995**, 28, 509-520, https://doi.org/10.1021/ar00060a007.

5. Korbukh, I.A., Abramova, L.N., Preobrazhenskaya, M.N. (1978). Anomers of 1-O-acetyl-2,3,5-tri-O-benzoyl-D-arabinose. In: Townsend, L.B., Stuart Tipson, R. (Eds.), Nucleic Acid Chem. Improved and new synthetic procedures, methods and techniques. John Wiley and Sons.

6. Reist, E.J., Goodman, L, Baker, B.R. Potential anticancer agents. VIII. Synthesis of nucleosides derived from L-talofuranose. *J. Am. Chem. Soc*. **1958**, 80, 5775-5779.

7. Prasad, A.K.; Kumar, V.; Malhotra, S.; Ravikumar, V.T.; Sanghvi, Y.S.; Parmar, V.S. ‘Green’ methodology for efficient and selective benzoylation of nucleosides using benzoyl cyanide in an ionic liquid. *Bioorg. Med. Chem*. **2005**, 13, 4467-4472, https://doi.org/10.1016/j.bmc.2005.04.038.

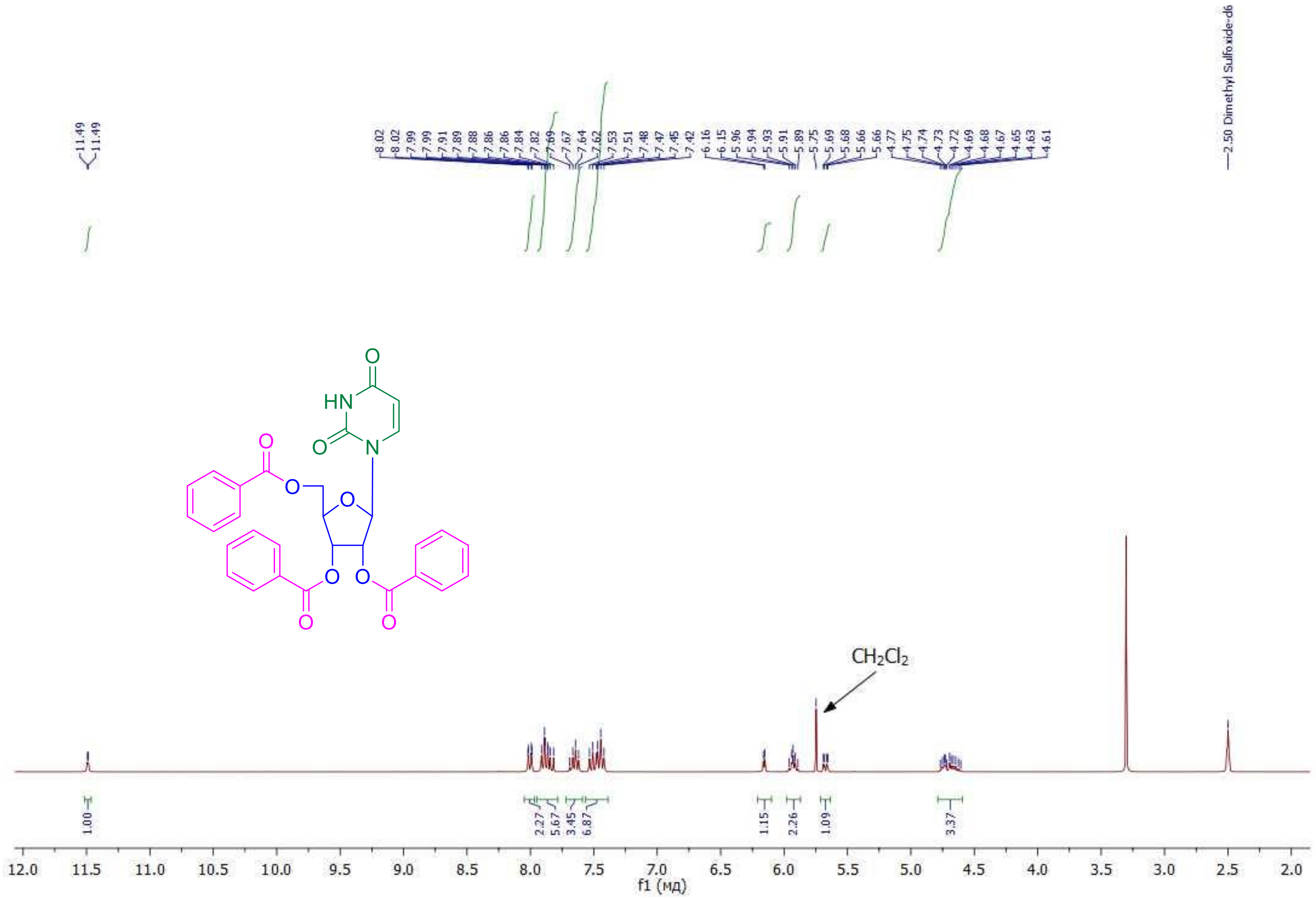

Fig.S1. ^{1}H-NMR-spectrum (300 MHz) of 2’,3’,5’-tri-*O*-benzoyl-D-uridine (**3D**) in DMSO-d6 at 298 K.

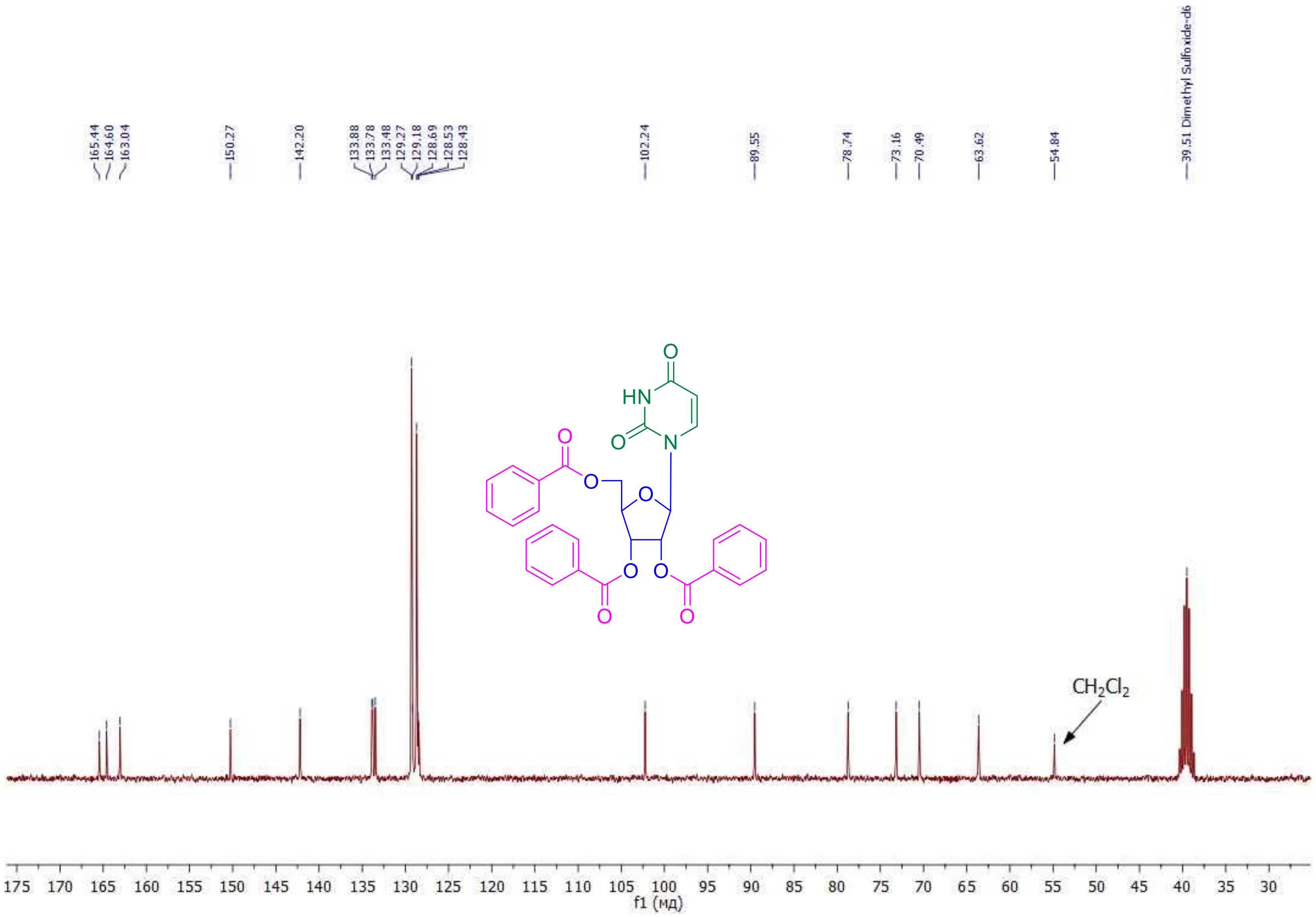

Fig.S2. ^{13}C-NMR-spectrum (75 MHz) 2’,3’,5’-tri-*O*-benzoyl-D-uridine (**3D**) in DMSO-d6 at 298 K.

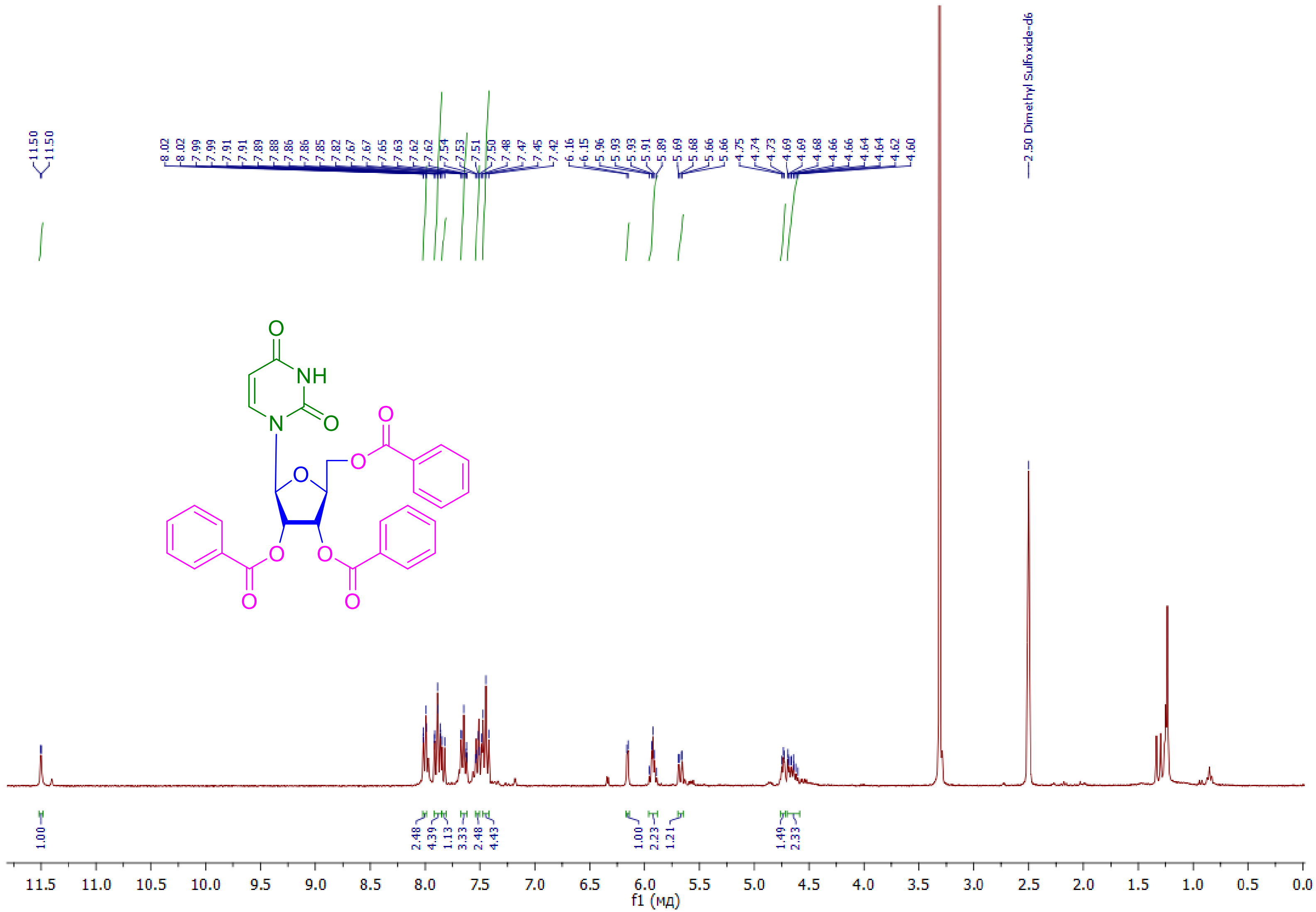

Fig.S3. ^{1}H-NMR-spectrum (300 MHz) of 2′,3′,5′-tri-*O*-benzoyl-L-uridine *(***3L***)* in DMSO-d_6 at 298 K

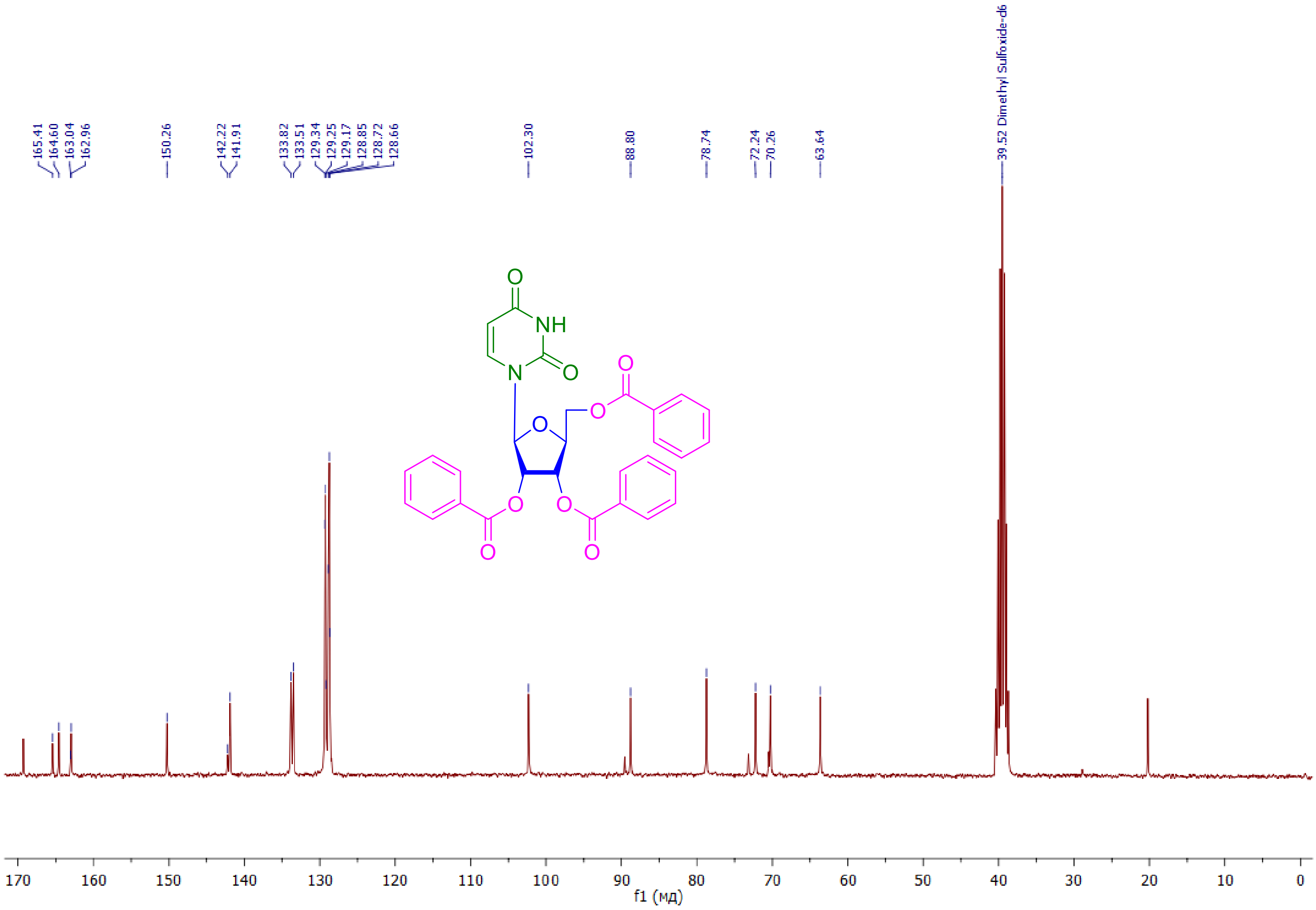

Fig.S4. ^{13}C-NMR-spectrum (75 MHz) of L-2′,3′,5′-tri-*O*-benzoyluridine ***(3L)*** in DMSO-d_6 at 298 K

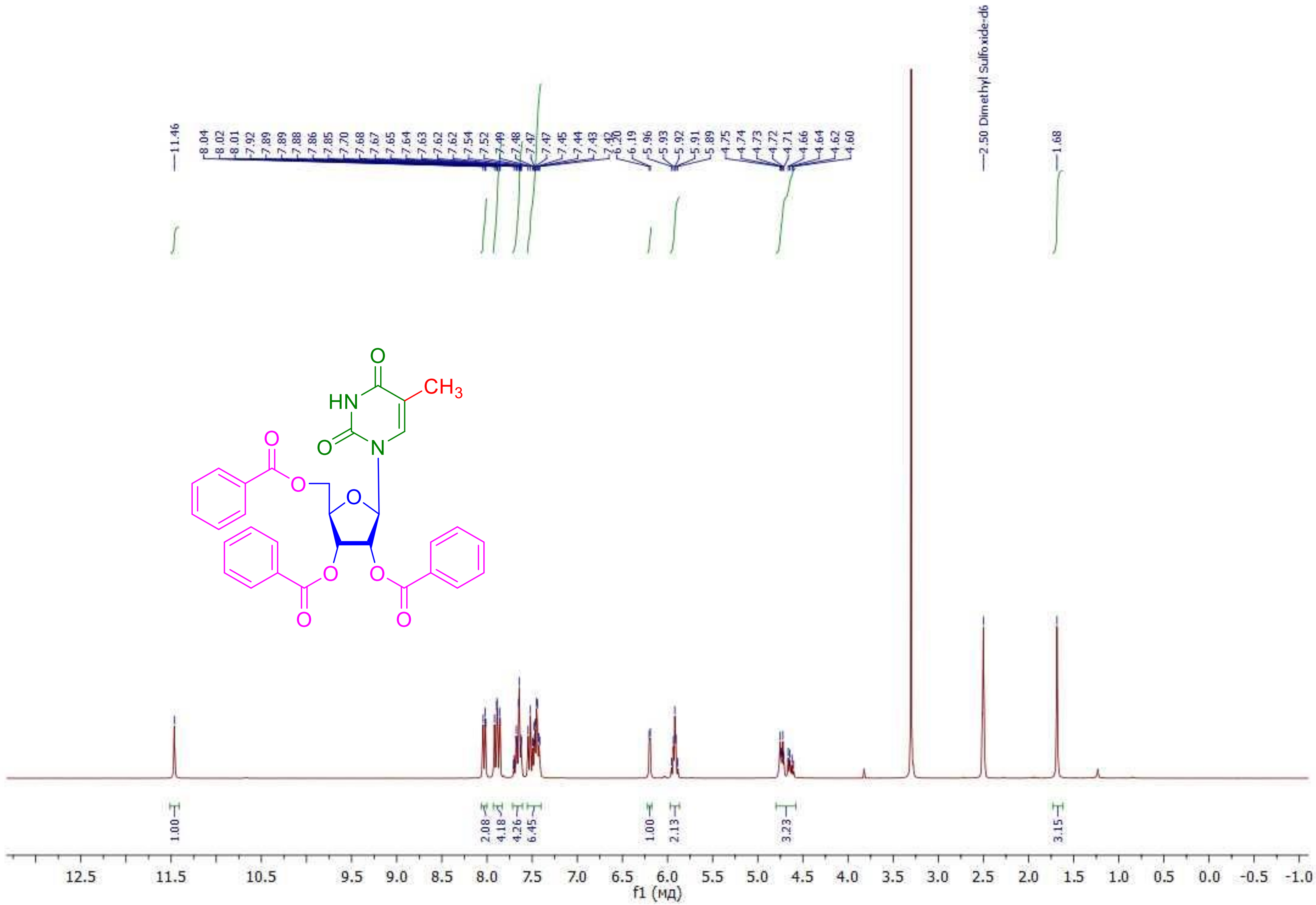

Fig.S5. ^{1}H-NMR-spectrum (300 MHz) of 2′,3′,5′-tri-O-benzoyl-5-methyl-D-uridine (**4D**) in DMSO-d6 at 298 K.

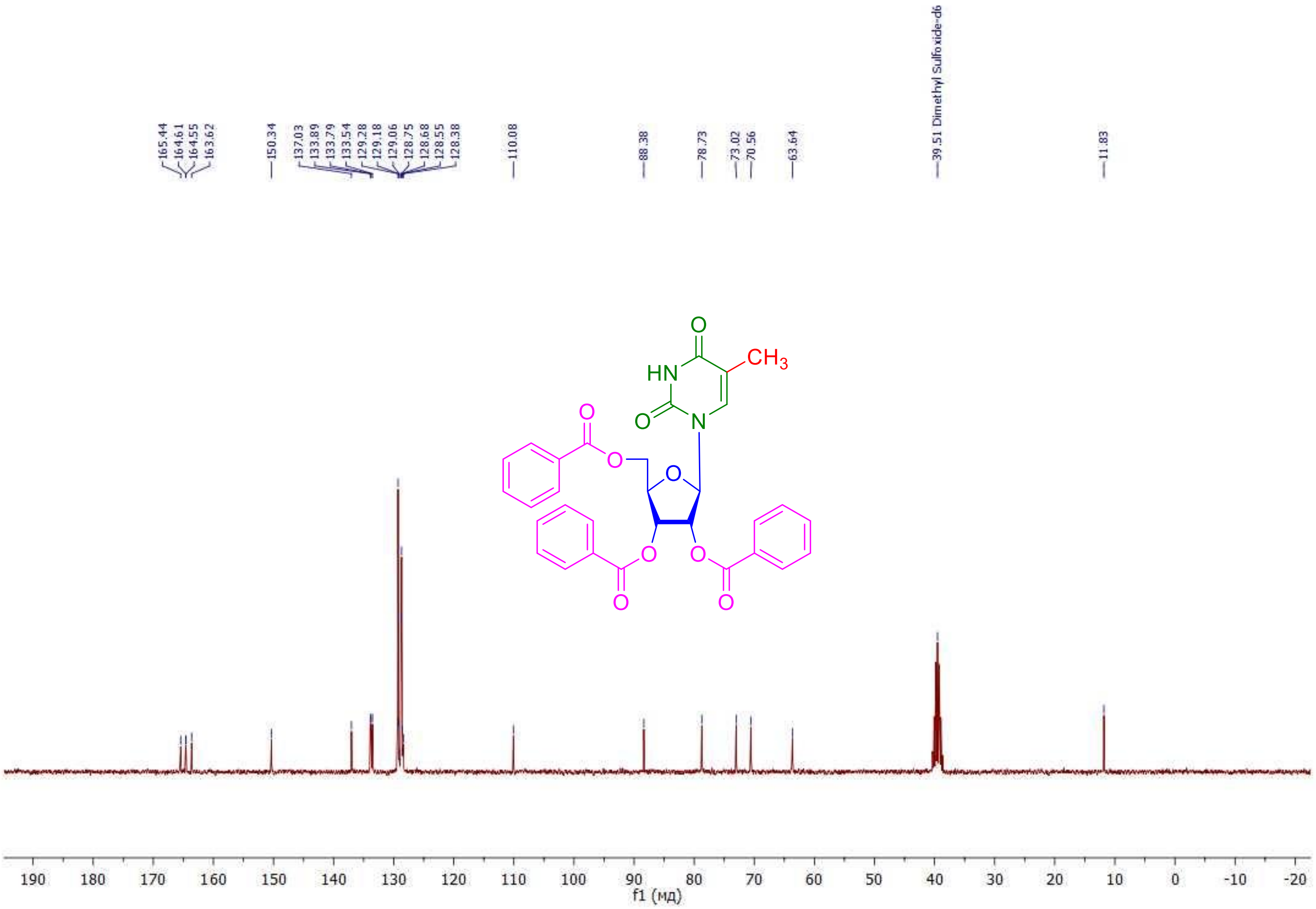

Fig.S6. ^{13}C-NMR-spectrum (75 MHz) of of 2′,3′,5′-tri-O-benzoyl-5-methyl-D-uridine (**4D**) in DMSO-d6 at 298 K.

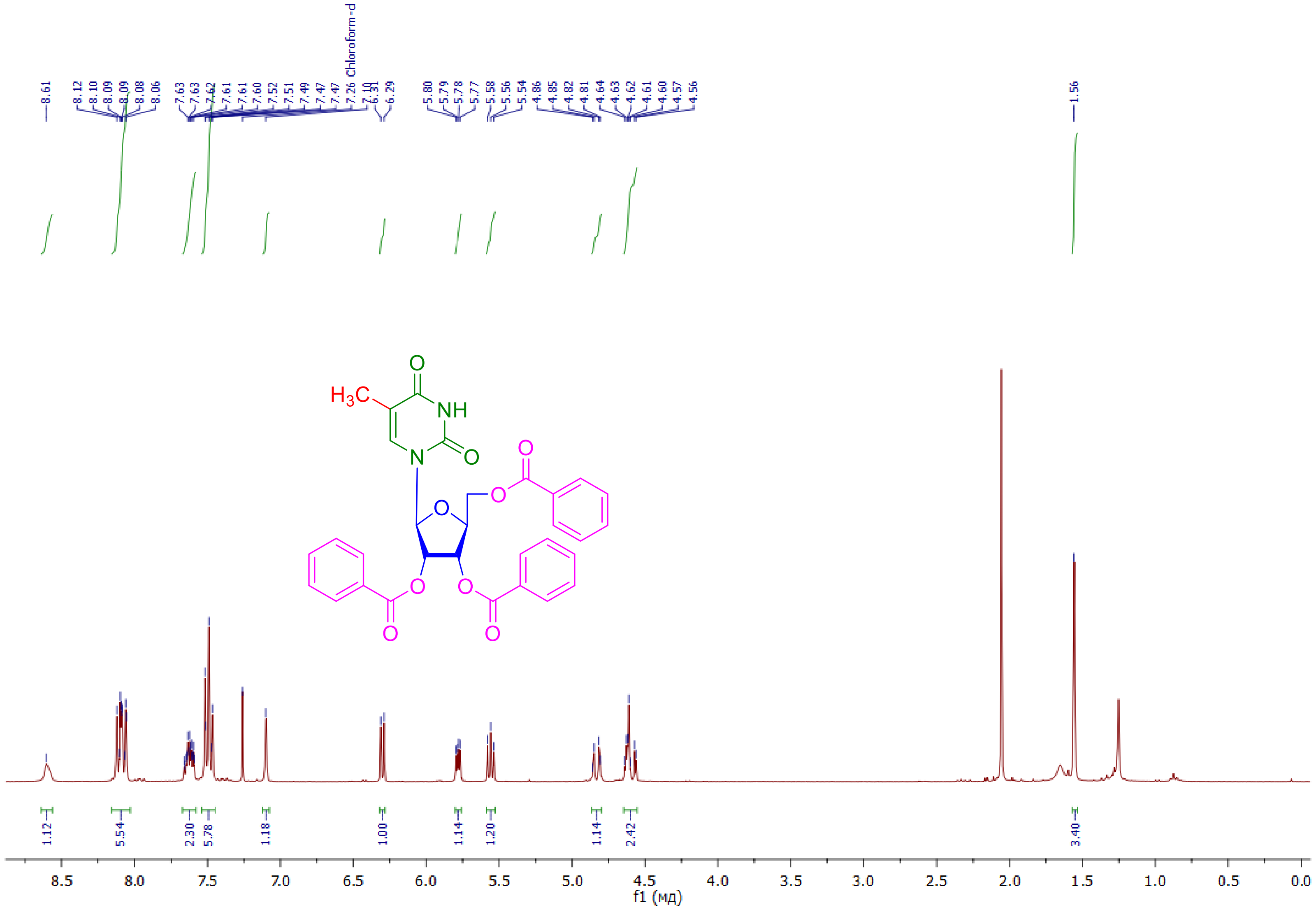

Fig.S7. ^{1}H-NMR-spectrum (300 MHz) of 2′,3′,5′-tri-O-benzoyl-L-ribothymidine ***(4L)*** in $CDCl_3$ at 298 K.

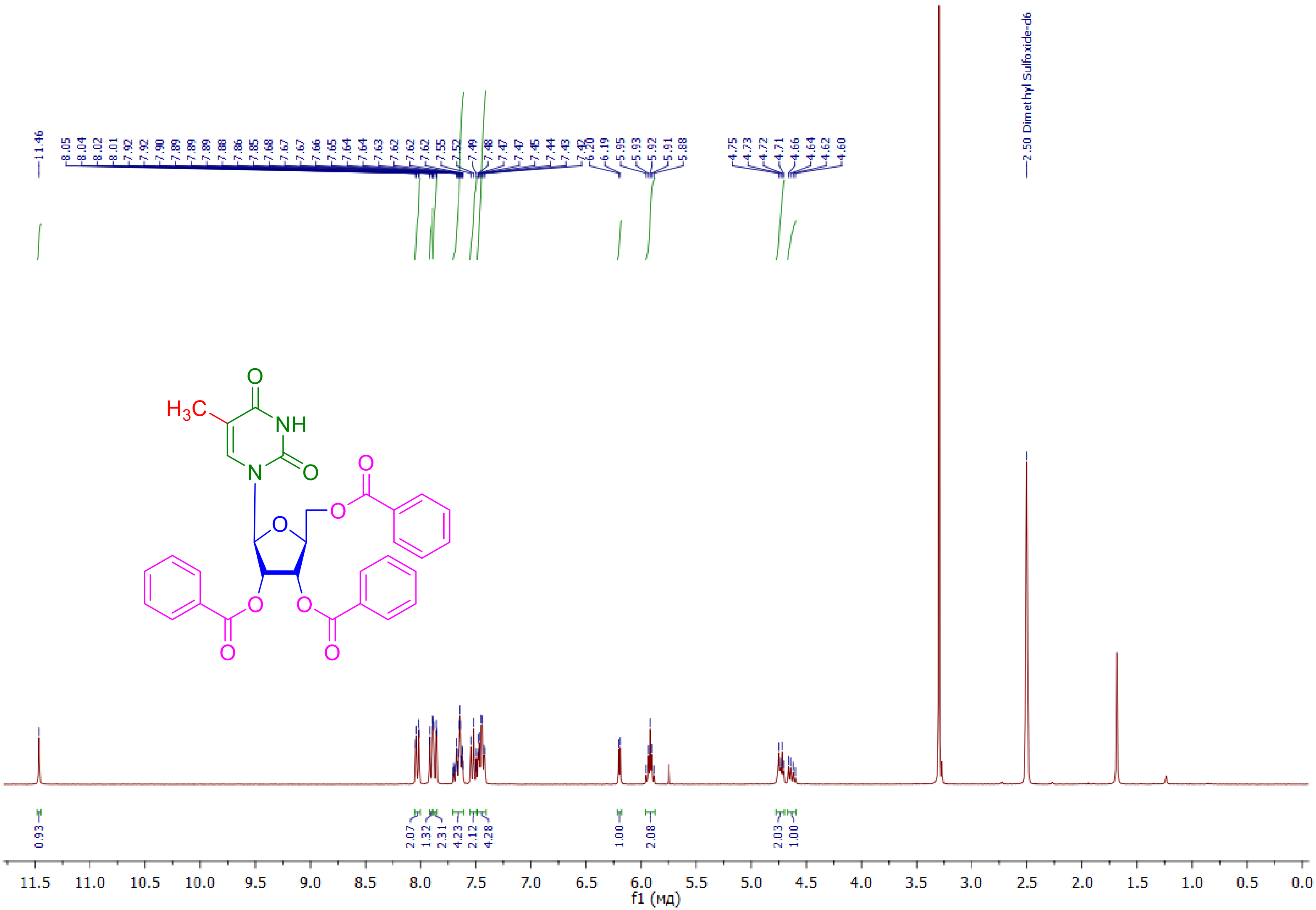

Fig.S8. ^{1}H-NMR-spectrum (300 MHz) of 2′,3′,5′-tri-O-benzoyl-L-ribothymidine ***(4L)*** in DMSO-d_6 at 298 K.

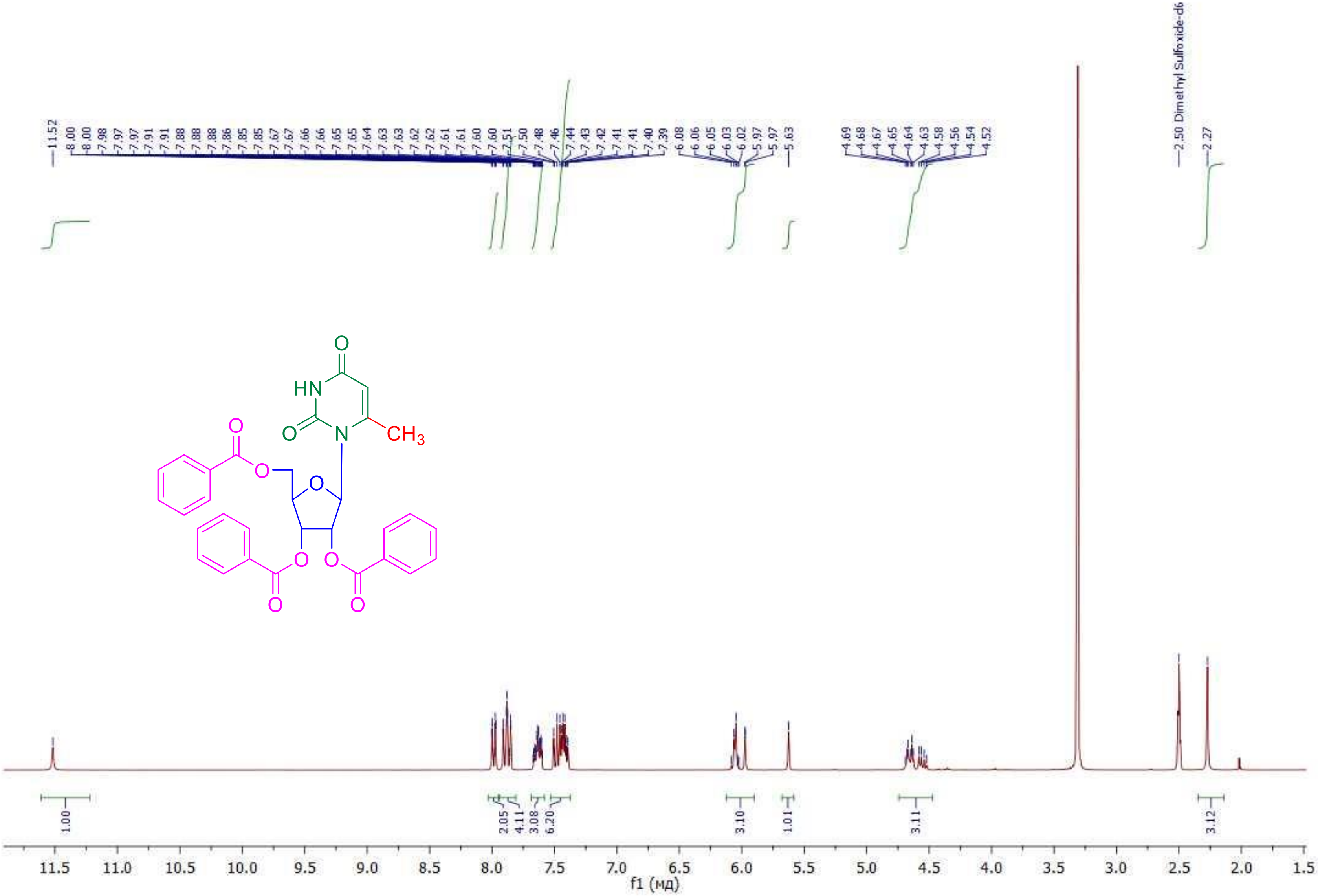

Fig.S9. ^{1}H-NMR-spectrum (75 MHz) of 2′,3′,5′-tri-O-benzoyl-6-methyl-D-uridine (**4′D**) in DMSO-d6 at 298 K.

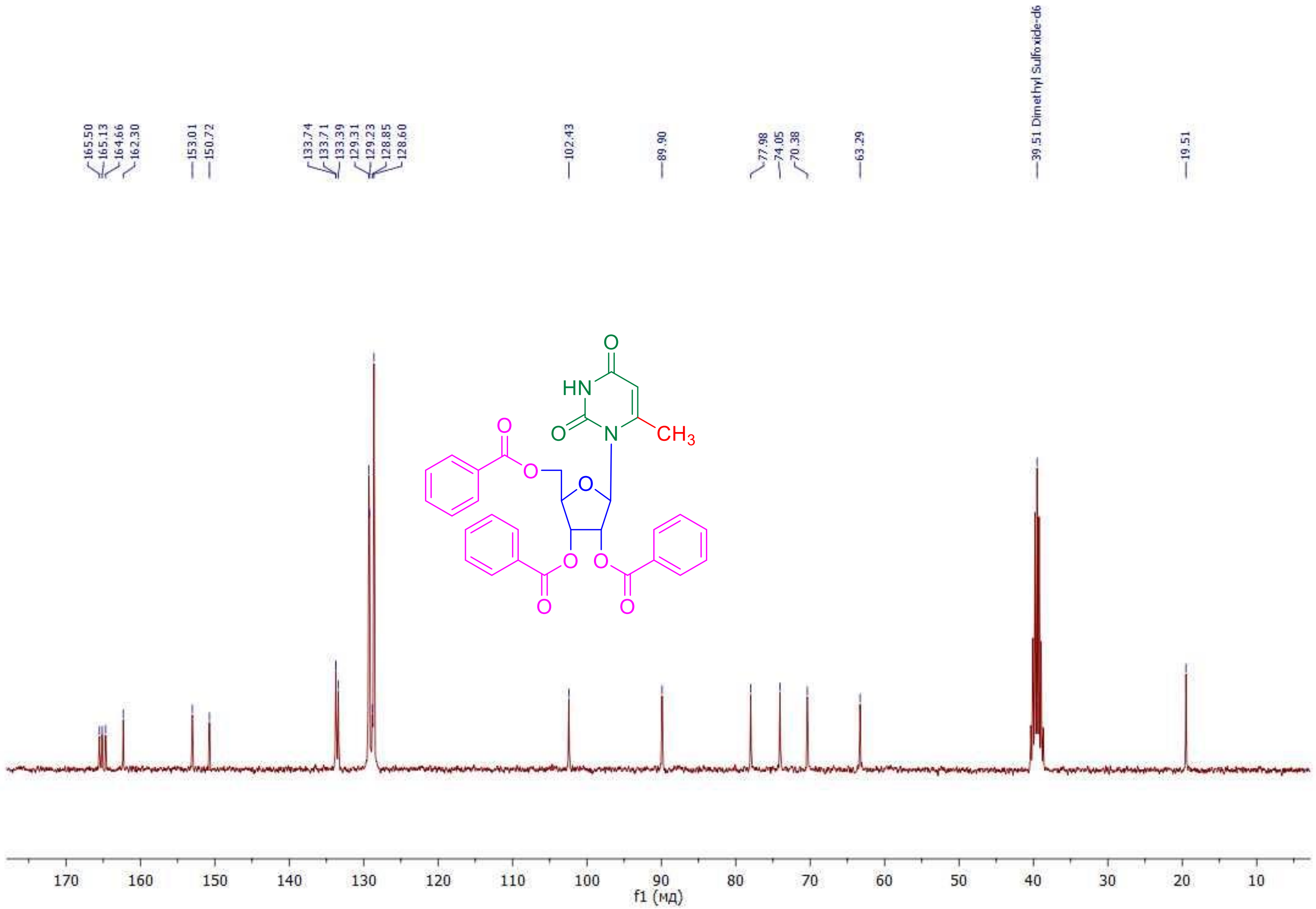

Fig.S10. ^{13}C-NMR-spectrum (75 MHz) of 2′,3′,5′-*tri*-O-benzoyl-6-methyl-D-uridine ***(4′D)*** in DMSO-d6 at 298 K.

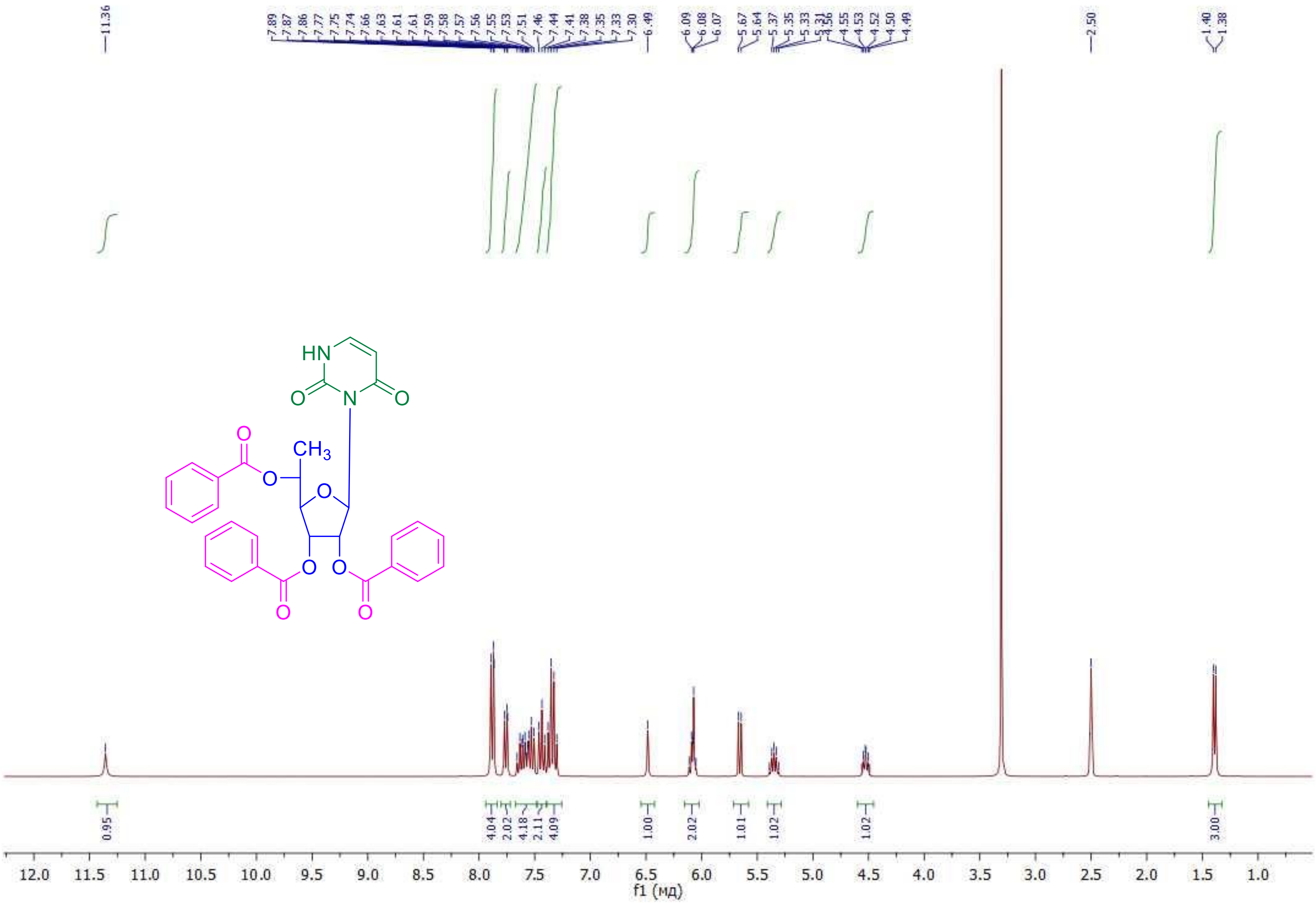

Fig.S11. ^{1}H-NMR-spectrum (300 MHz) of 3-[5(*S*)-C-methyl-2,3,5-tri-*O*-benzoyl-β-D-ribofuranosyl]uracil (**5S**) in DMSO-d6 at 298 K.

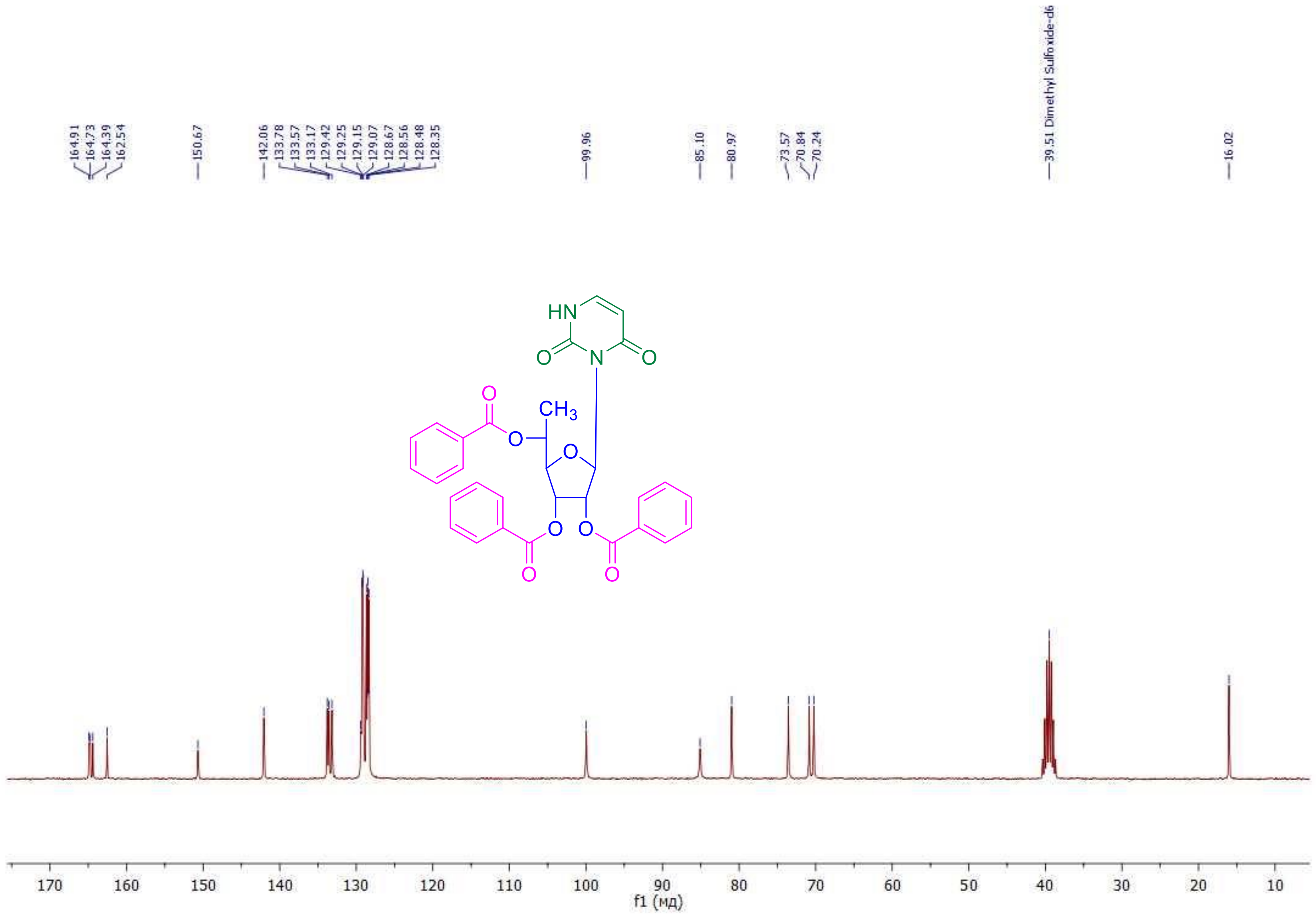

Fig.S12. ^{13}C-NMR-spectrum (75 MHz) of 3-[5(*S*)-*C*-methyl-2,3,5-tri-O-benzoyl-β-D-ribofuranosyl]uracil ***(5S)*** in DMSO-d6 at 298 K.

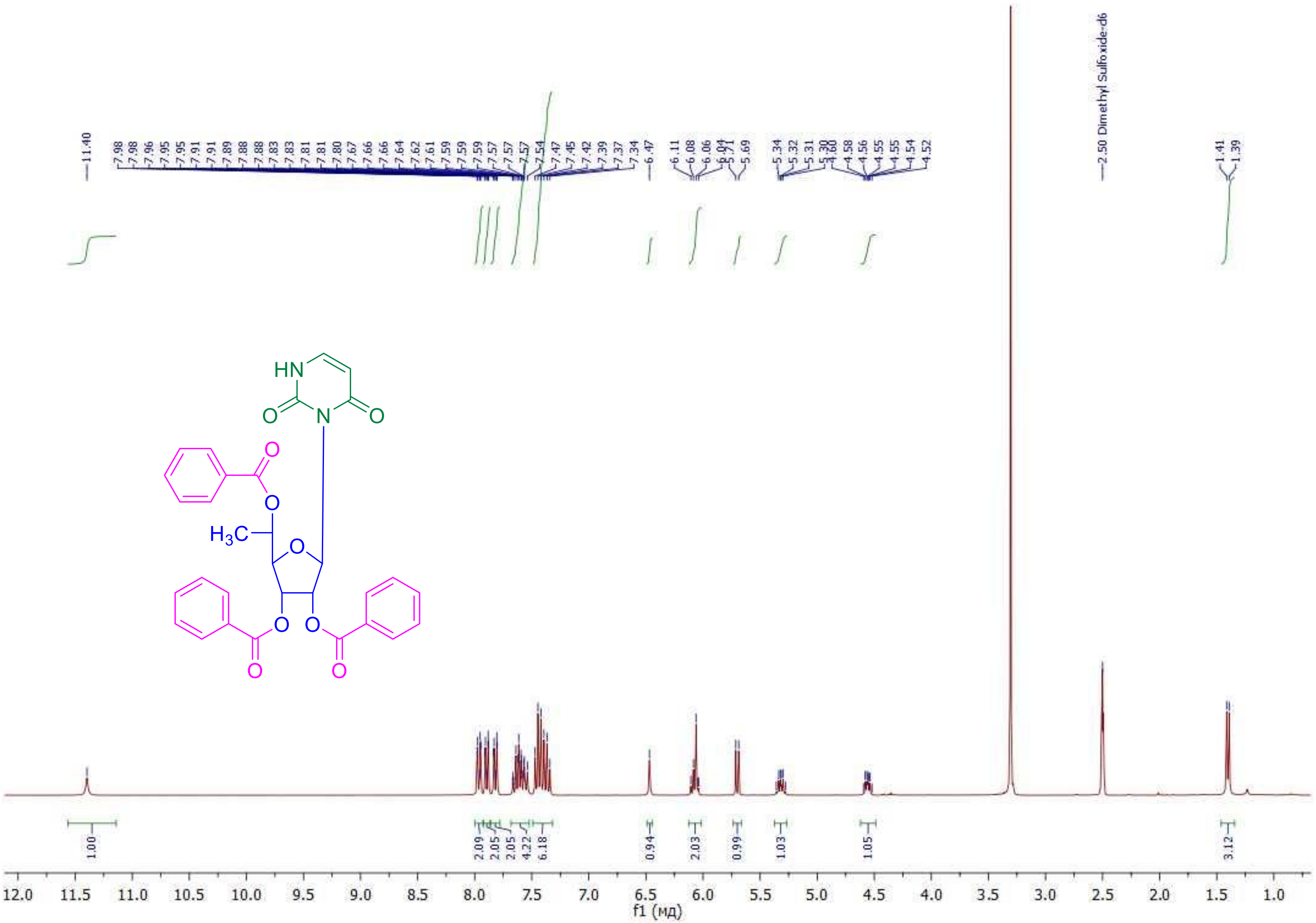

Fig.S13. ^{1}H-NMR-spectrum (300 MHz) of 3-[5(*R*)-*C*-methyl-2,3,5-O-tribenzoyl-β-D-ribofuranosyl]uracil (**5R**) in DMSO-d6 at 298 K.

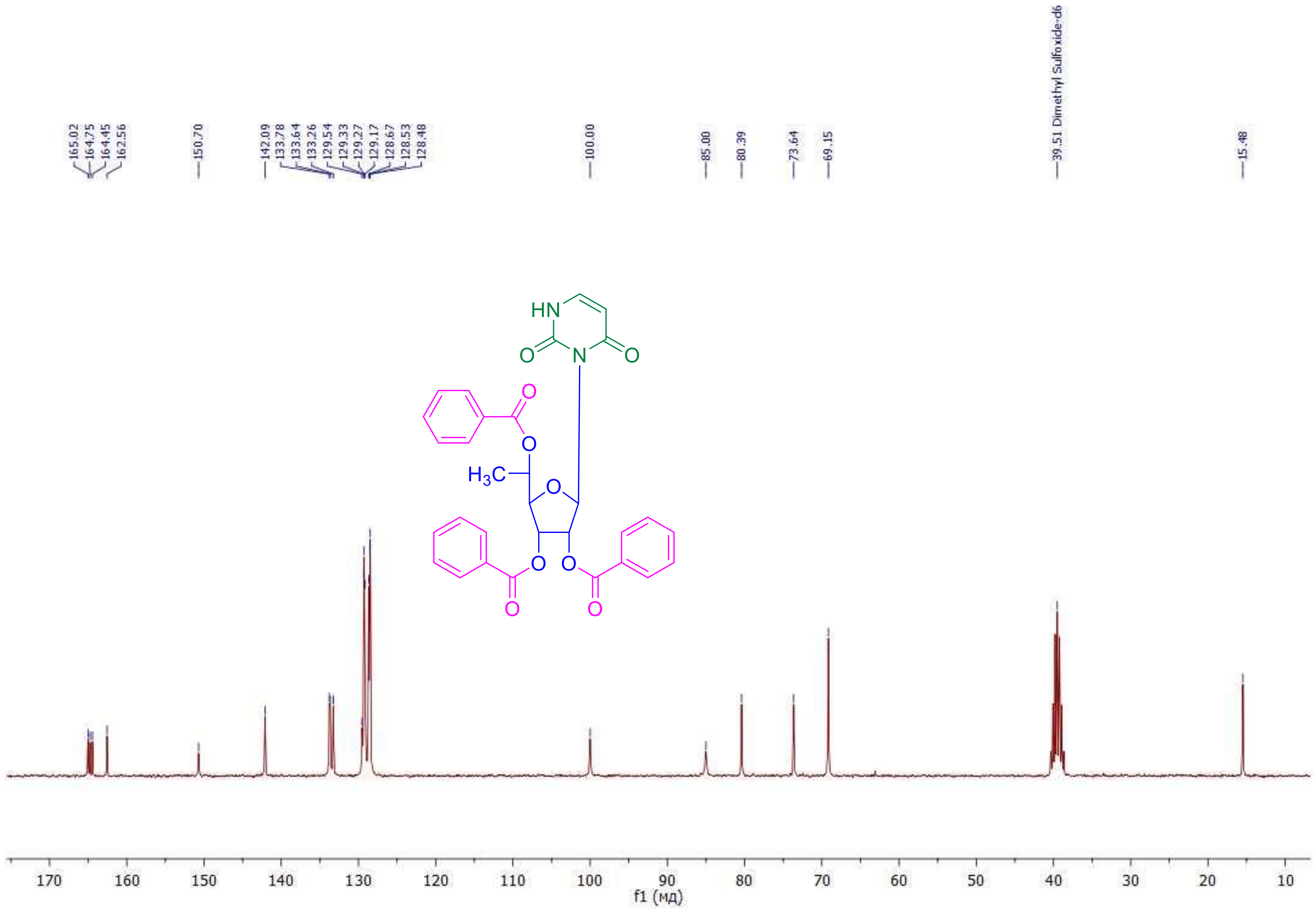

Fig.S14. ^{13}C-NMR-spectrum (75 MHz) of 3-[5(*R*)-*C*-methyl-2,3,5-O-tribenzoyl-β-D-ribofuranosyl]uracil ***(5R)*** in DMSO-d6 at 298 K.

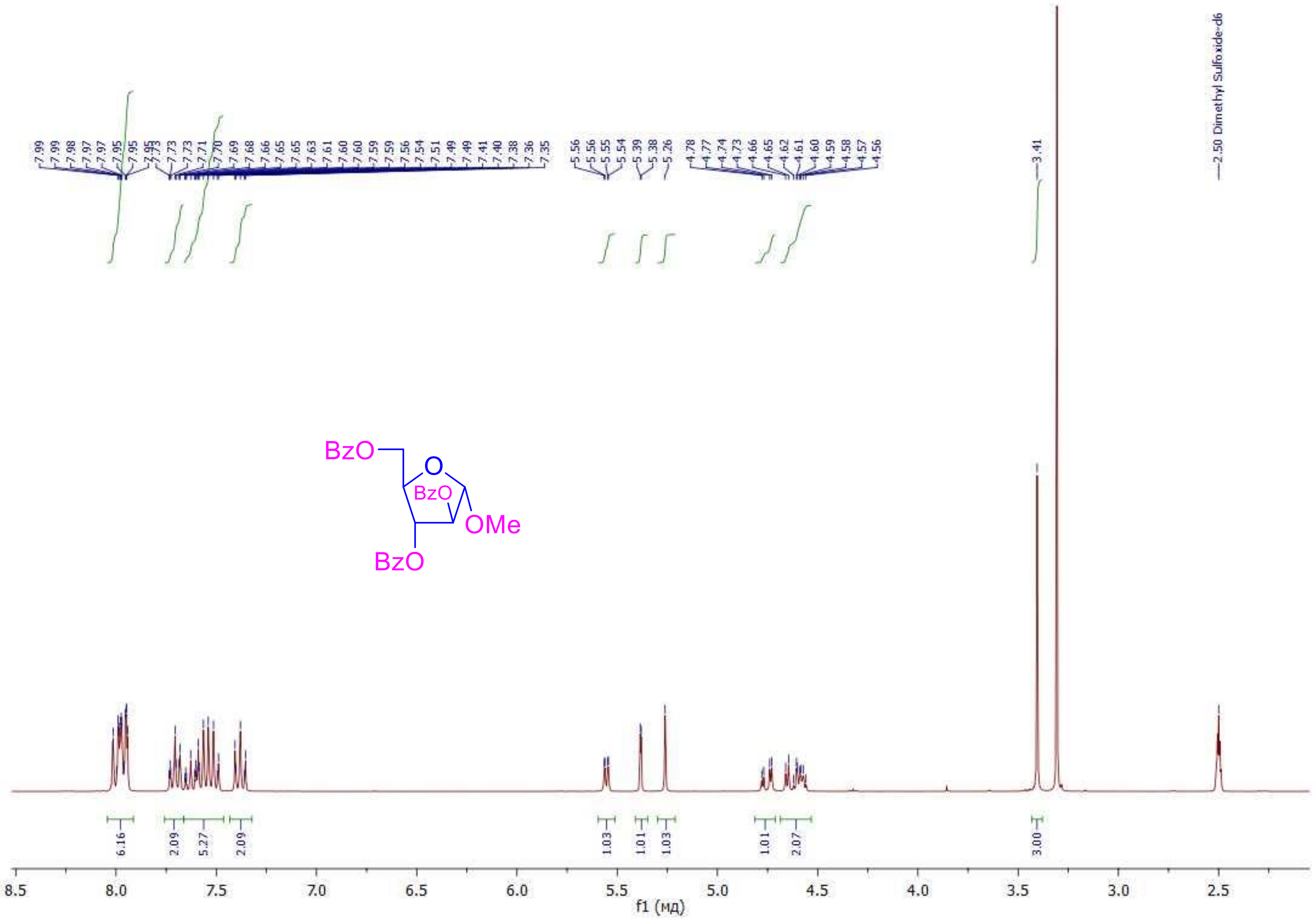

Fig.S15. ^{1}H-NMR-spectrum (300 MHz) of 1-*O*-methyl-2,3,5-tri-*O*-benzoyl-α-D-arabinofuranose (**1D**) in DMSO-d6 at 298 K.

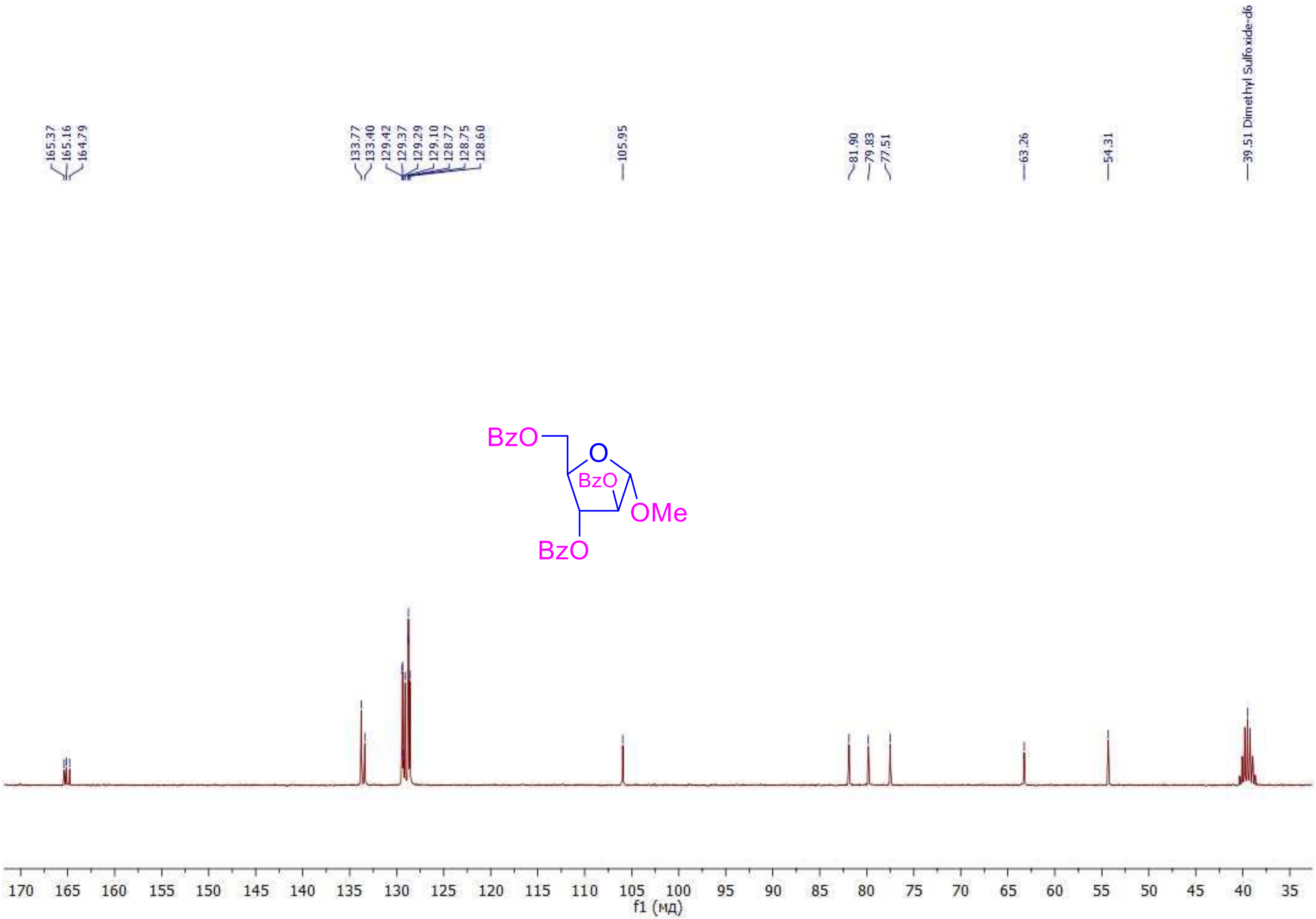

Fig.S16. ^{13}C-NMR-spectrum (75 MHz) of 1-O-methyl-2,3,5-tri-O-benzoyl-α-D-arabinofuranose ***(1D)*** in DMSO-d6 at 298 K.

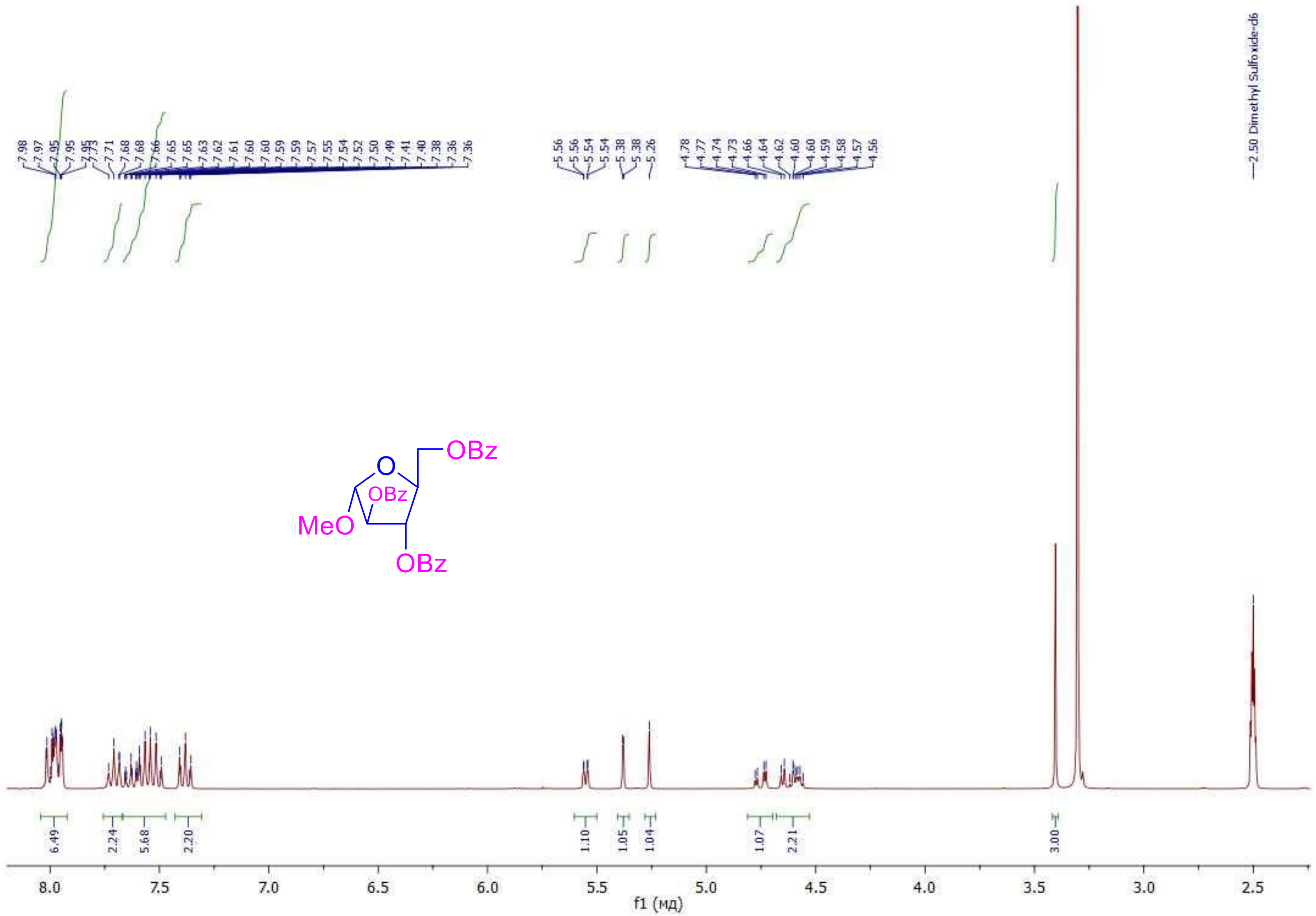

Fig.S17. ^{1}H-NMR-spectrum (300 MHz) of 1-*O*-methyl-2,3,5-tri-*O*-benzoyl-α-L-arabinofuranose (**1L**) in DMSO-d6 at 298 K.

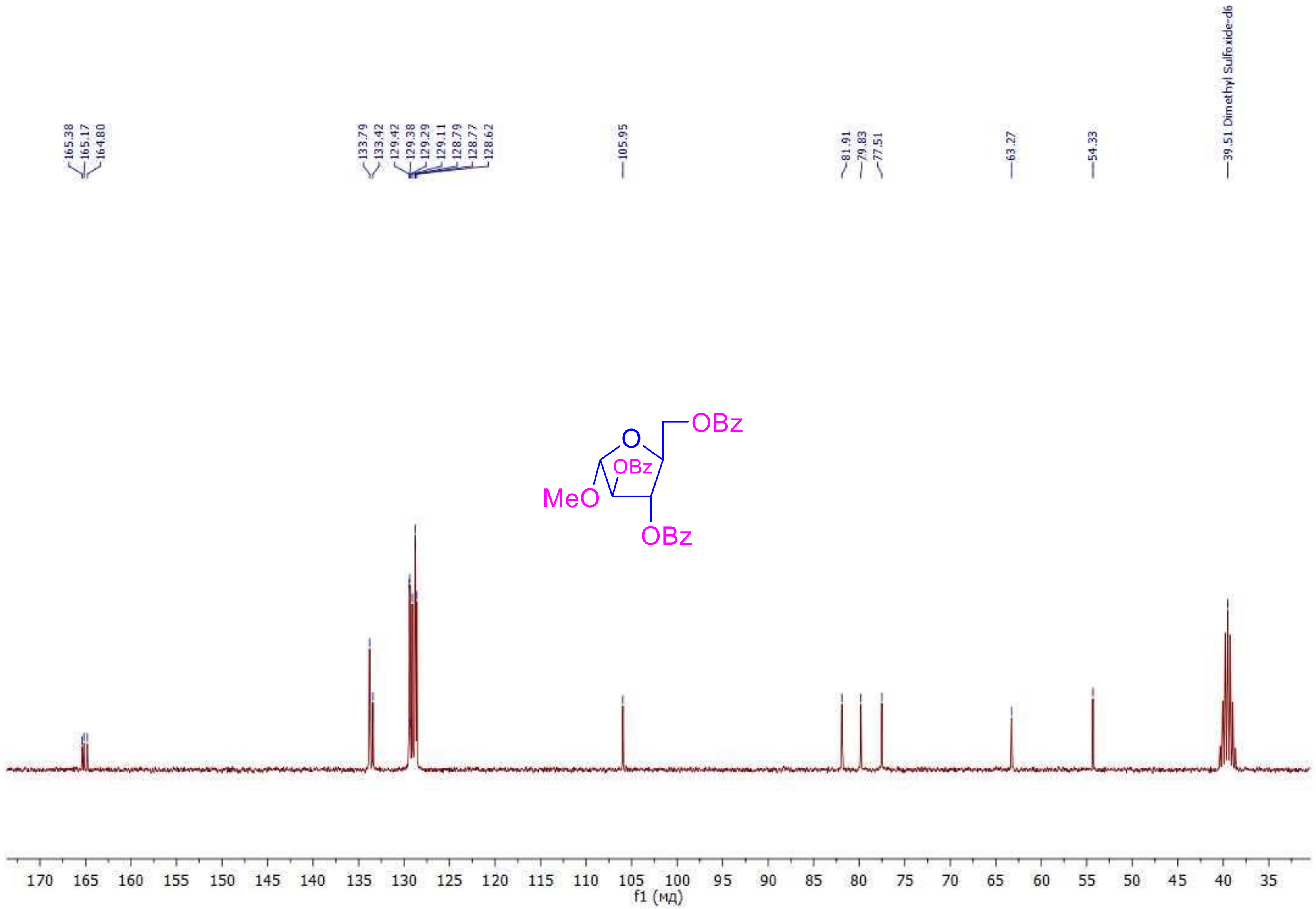

Fig.S18. ^{13}C-NMR-spectrum (75 MHz) of *1*-O-*methyl-2,3,5-tri*-O-*benzoyl-α-L-arabinofuranose* (***1L***) in DMSO-d6 at 298 K.

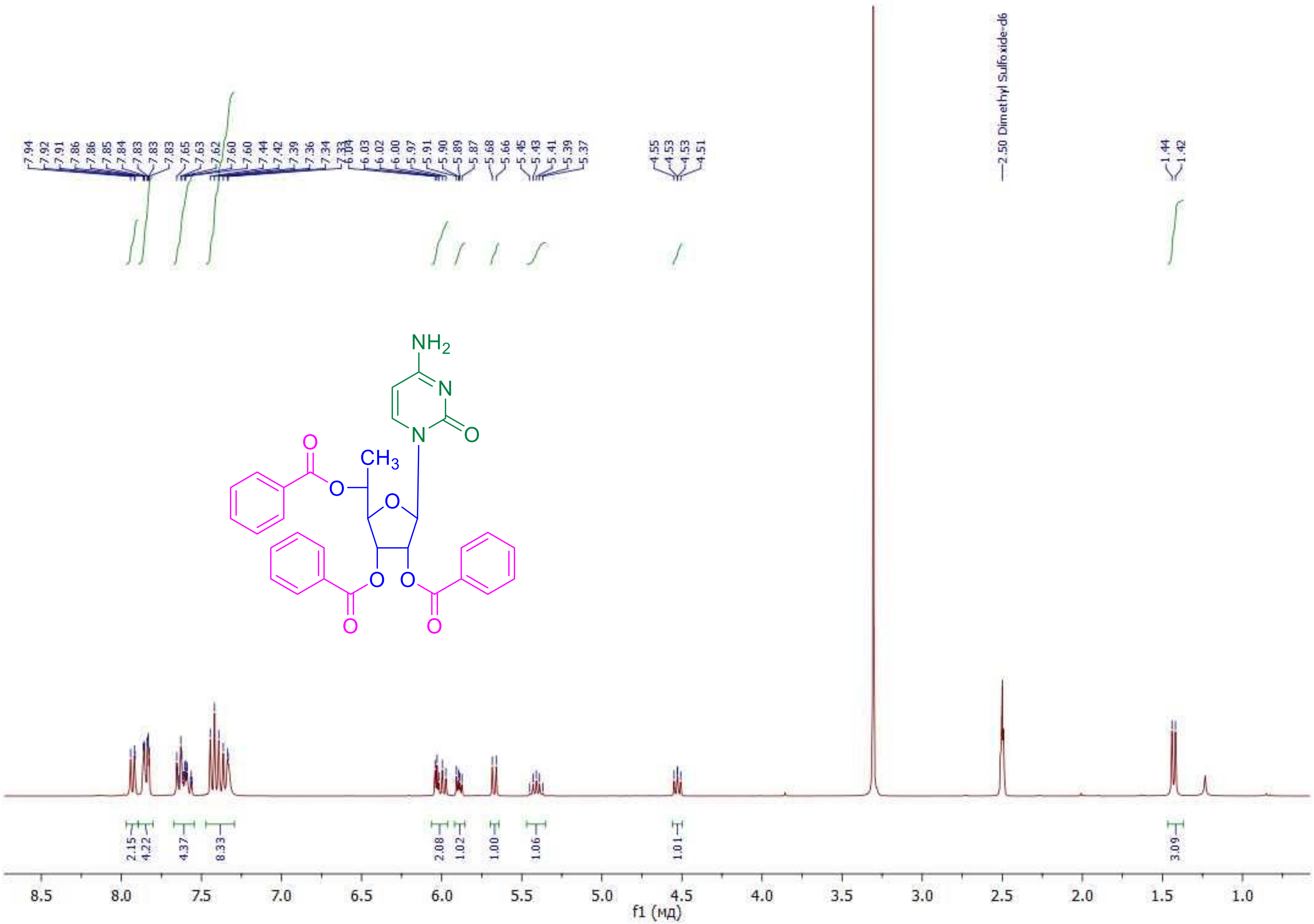

Fig.S19. ^{1}H-NMR-spectrum (300 MHz) of 1-[5(*S*)-*C*-methyl-2,3,5-tri-*O*-benzoyl-β-D-ribofuranosyl]cytosine (**6S**) in DMSO-d6 at 298 K.

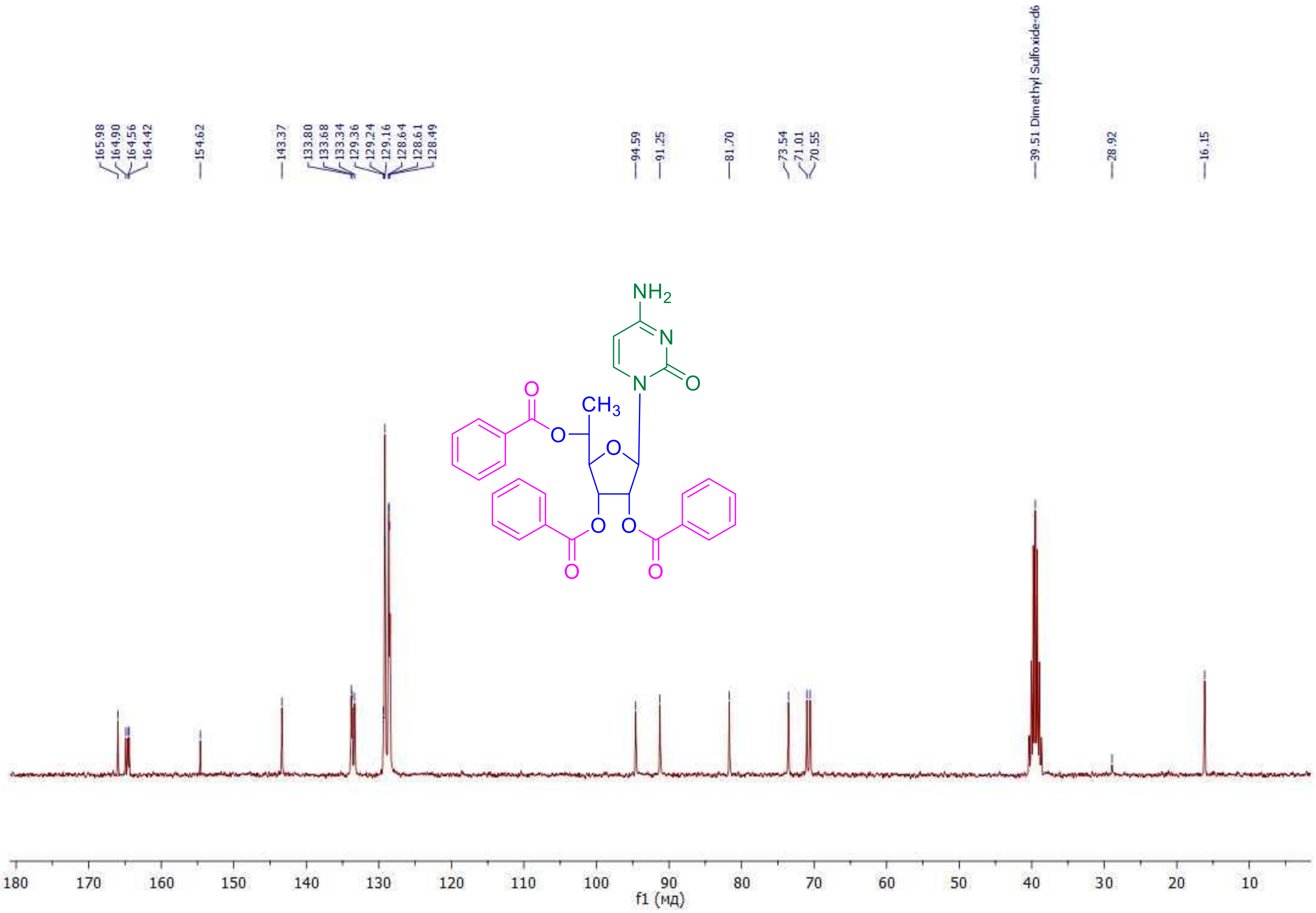

Fig.S20. ^{13}C-NMR-spectrum (75 MHz) of 1-[5(*S*)-*C*-methyl-2,3,5-tri-O-benzoyl-β-D-ribofuranosyl]cytosine ***(6S)*** in DMSO-d6 at 298 K.

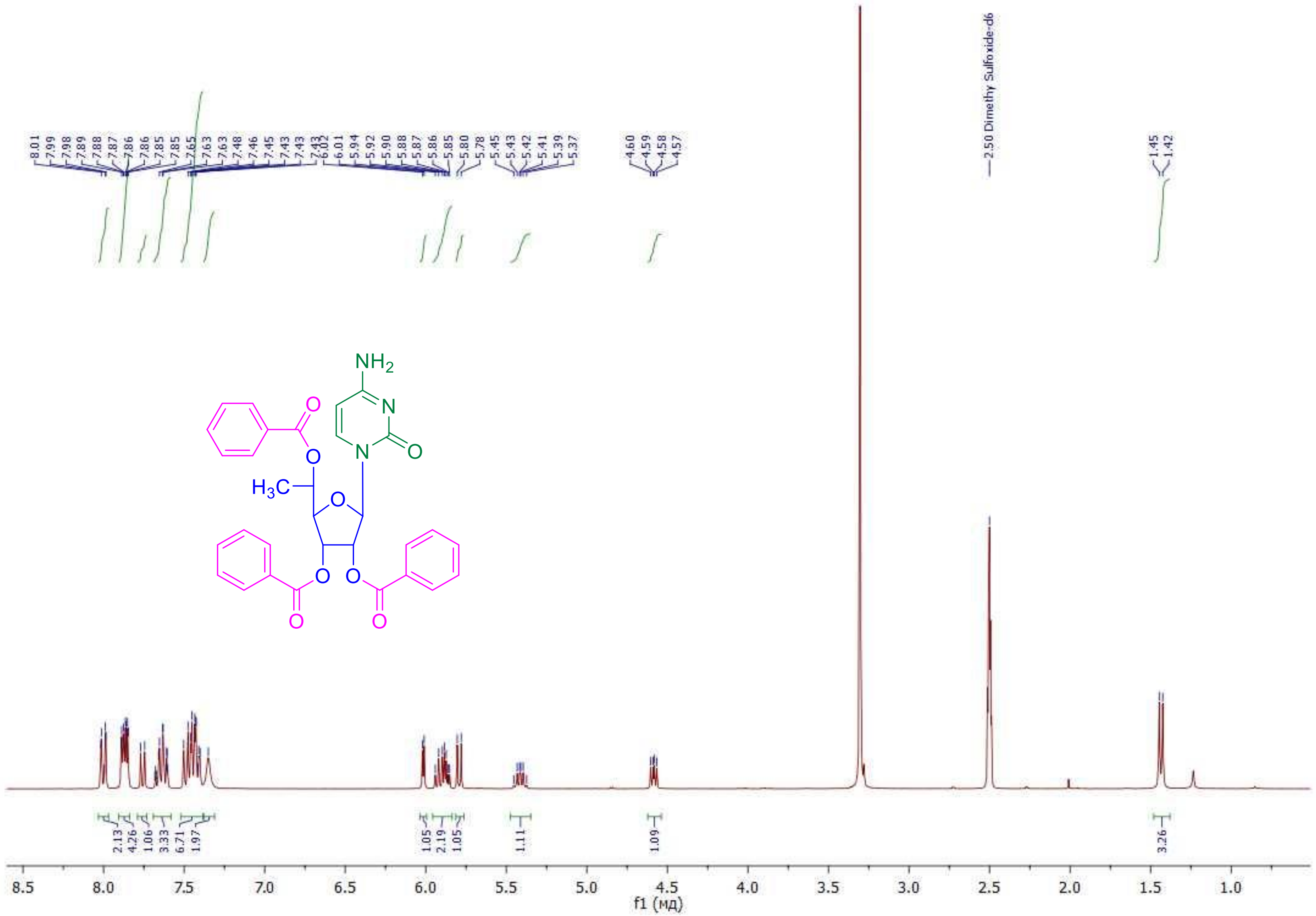

Fig.S21. ^{1}H-NMR-spectrum (300 MHz) of 1-[5(*R*)-*C*-methyl-2,3,5-tri-O-benzoyl-β-D-ribofuranosyl]cytosine (**6R**) in DMSO-d6 at 298 K.

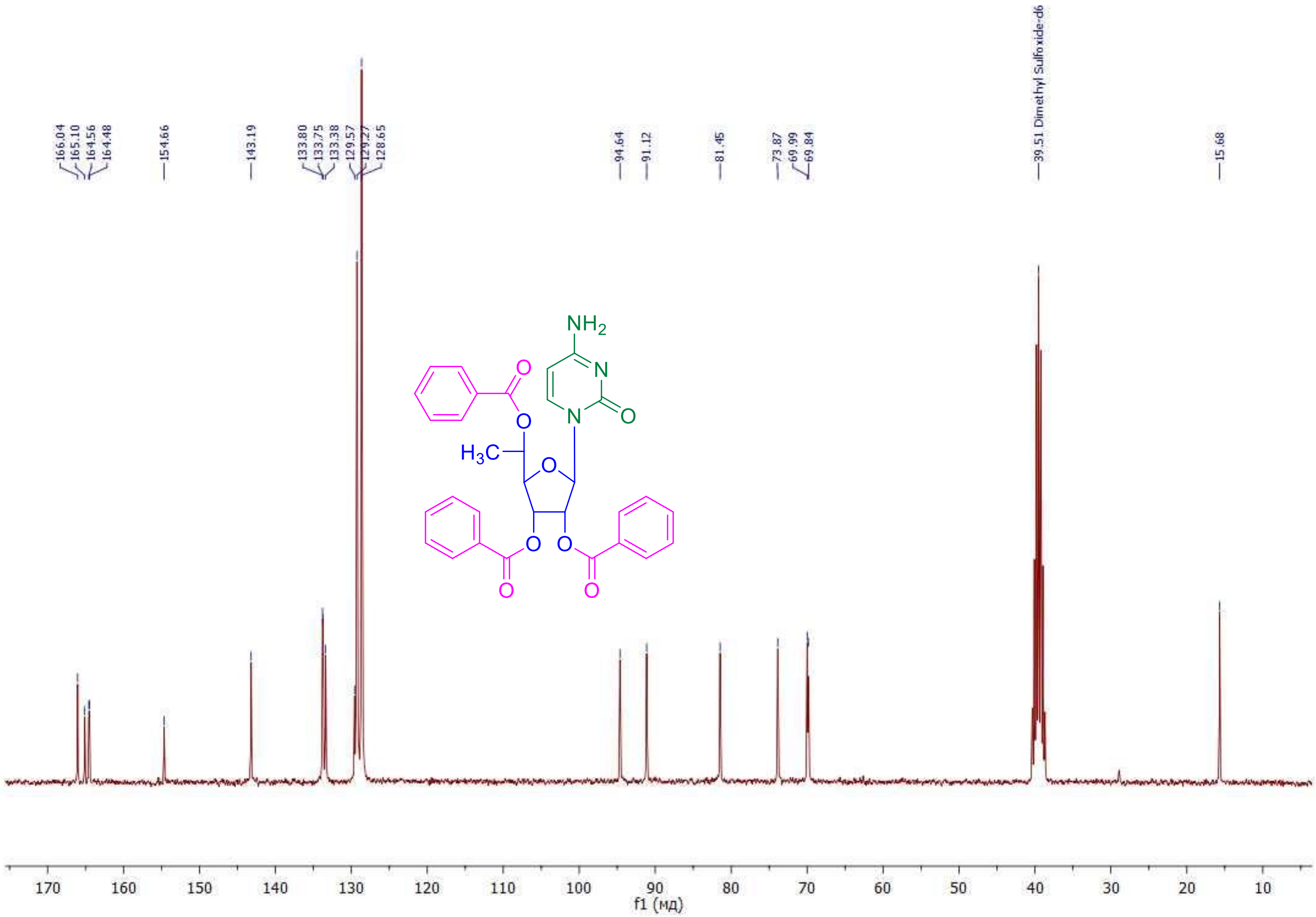

Fig.S22. ^{13}C-NMR-spectrum (75 MHz) of 1-[5(*R*)-*C*-methyl-2,3,5-tri-O-benzoyl-β-D-ribofuranosyl]cytosine ***(6R)*** in DMSO-d6 at 298 K.

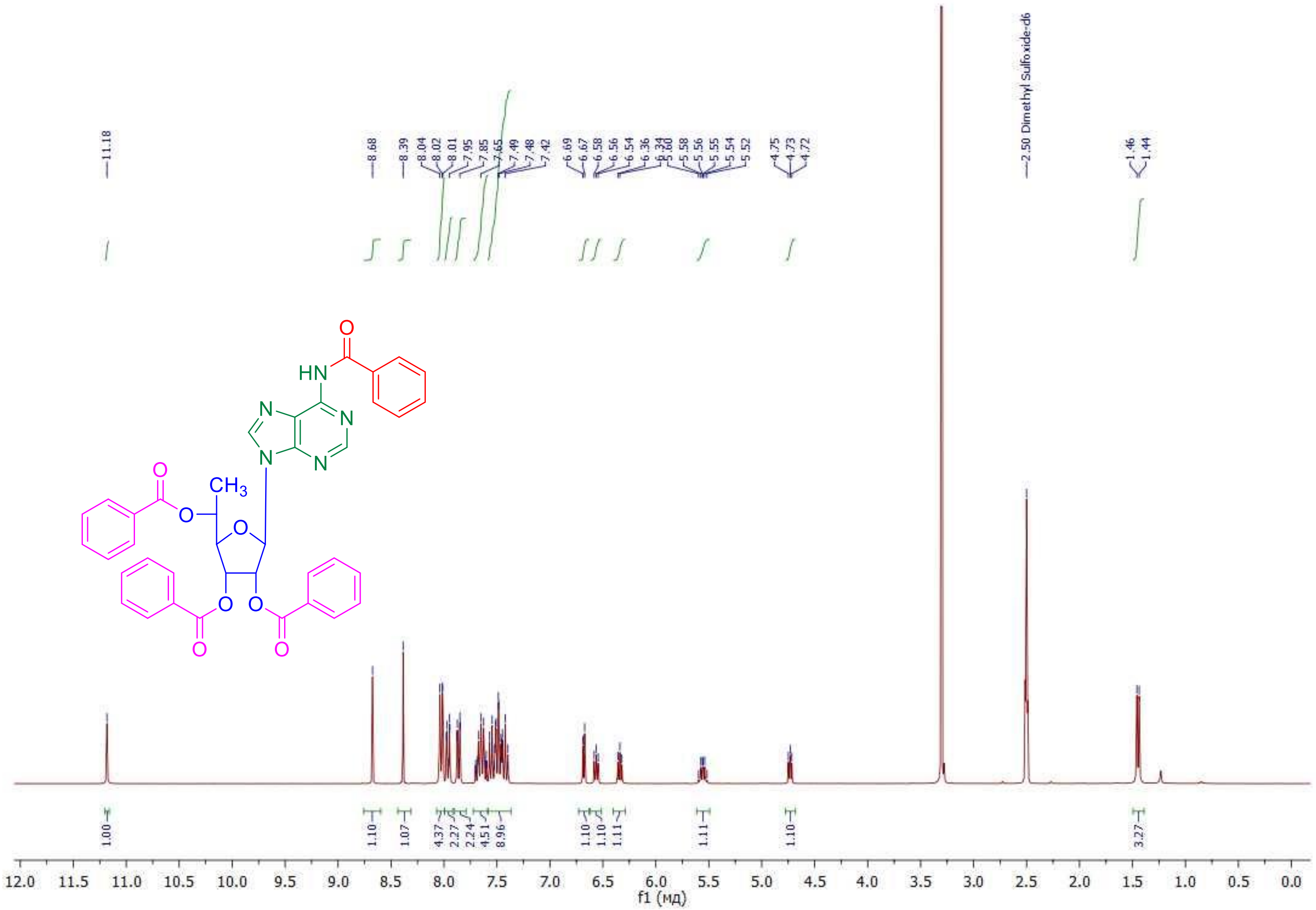

Fig.S23. ^{1}H-NMR-spectrum (300 MHz) of 5′(*S*)-*C*-methyl-2′,3′,5′-tri-*O*-benzoyl-*N*[6]-benzoyladenosine (**7S**) in DMSO-d6 at 298 K.

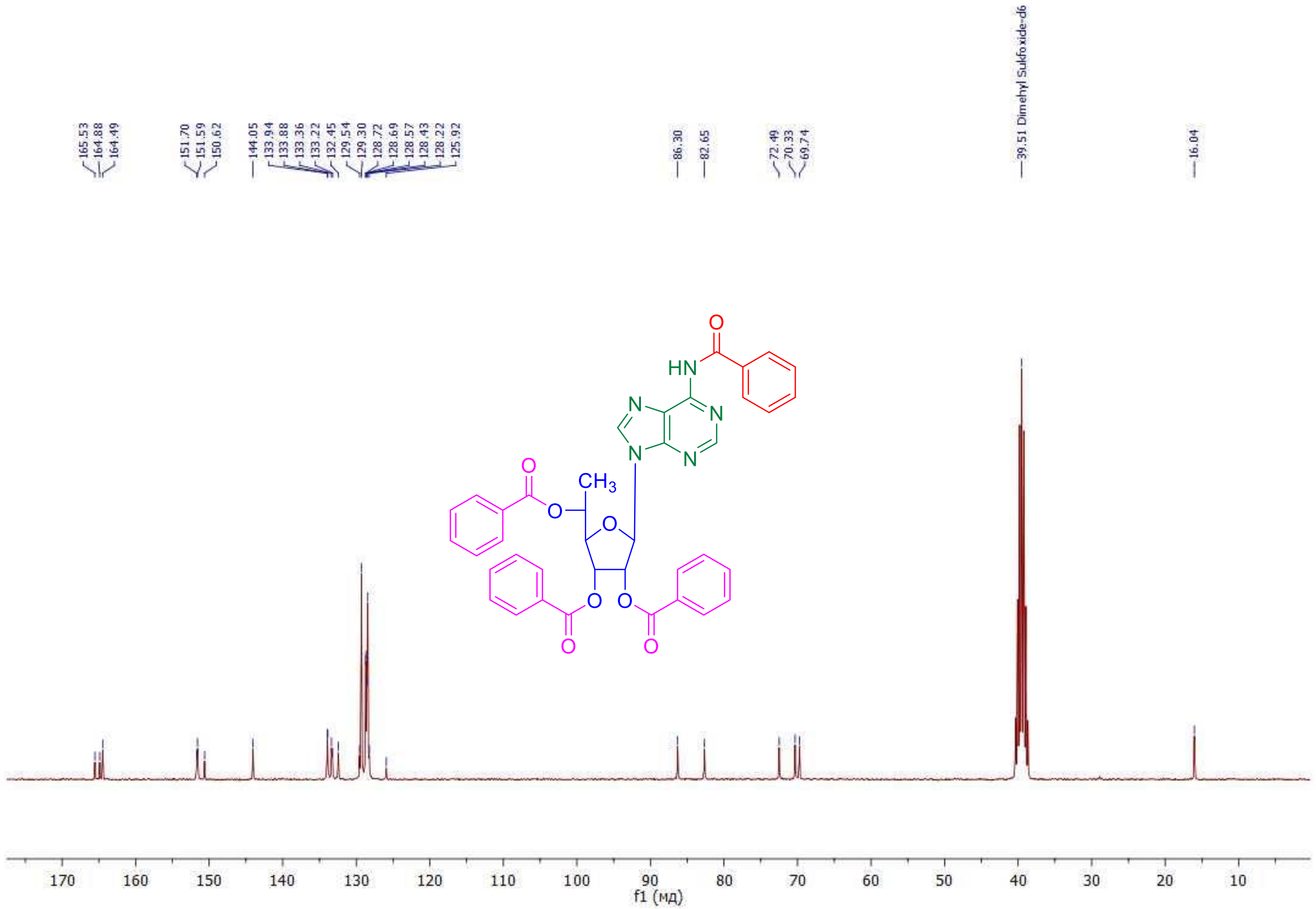

Fig.S24. ^{13}C-NMR-spectrum (75 MHz) of *5′(S)-C*-methyl-2′,3′,5′-tri-*O*-benzoyl-*N*^{6}-benzoyladenosine ***(7S)*** in DMSO-d6 at 298 K.

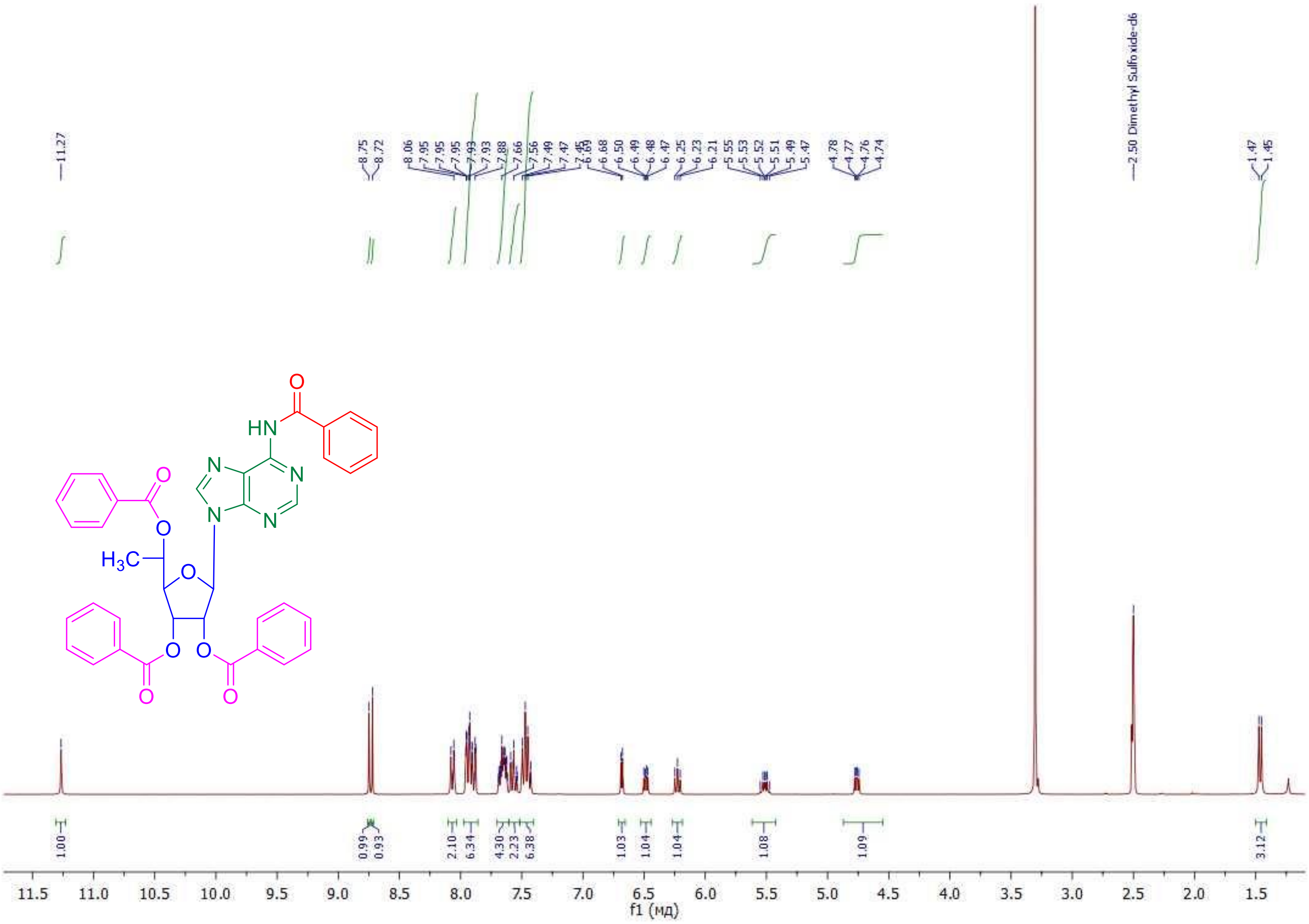

Fig.S25. ^{1}H-NMR-spectrum (300 MHz) of 5′(*R*)-*C*-methyl-2′,3′,5′-tri-*O*-benzoyl-*N*[6]-benzoyladenosine (**7R**) in DMSO-d6 at 298 K.

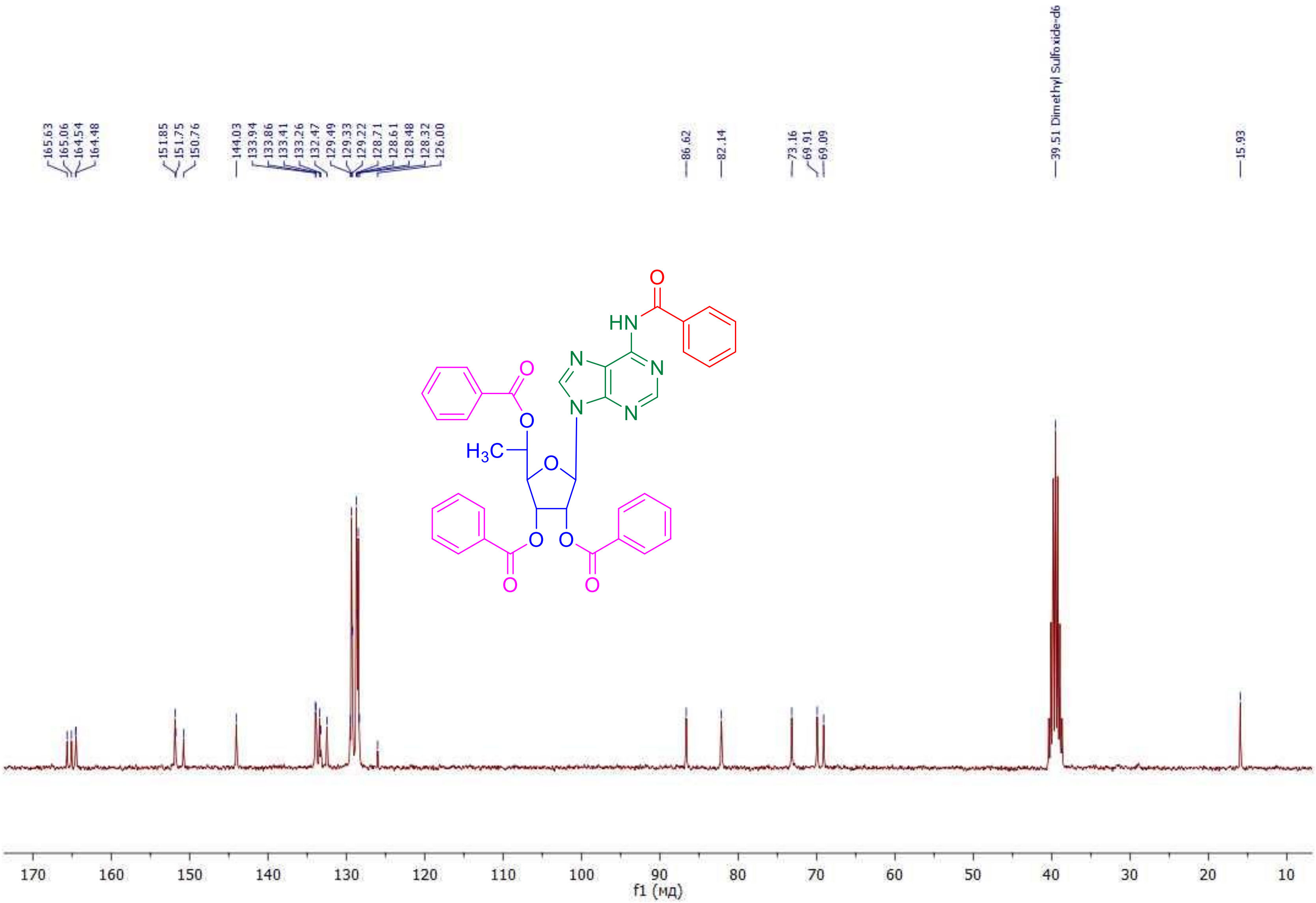

Fig.S26. ^{13}C-NMR-spectrum (75 MHz) of 5′(*R*)-*C*-methyl-2′,3′,5′-tri-O-benzoyl-*N*^{6}-benzoyladenosine ***(7R)*** in DMSO-d6 at 298 K.

Fig. S27. Selectivity of nucleoside stereoisomers

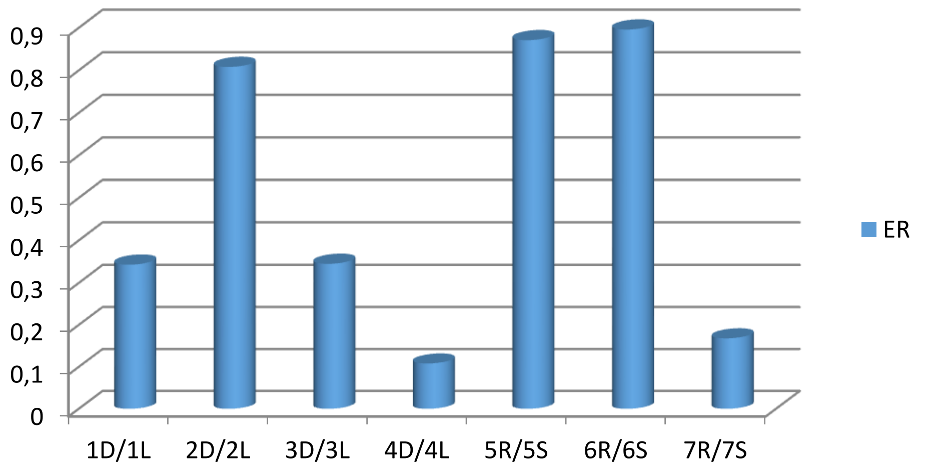

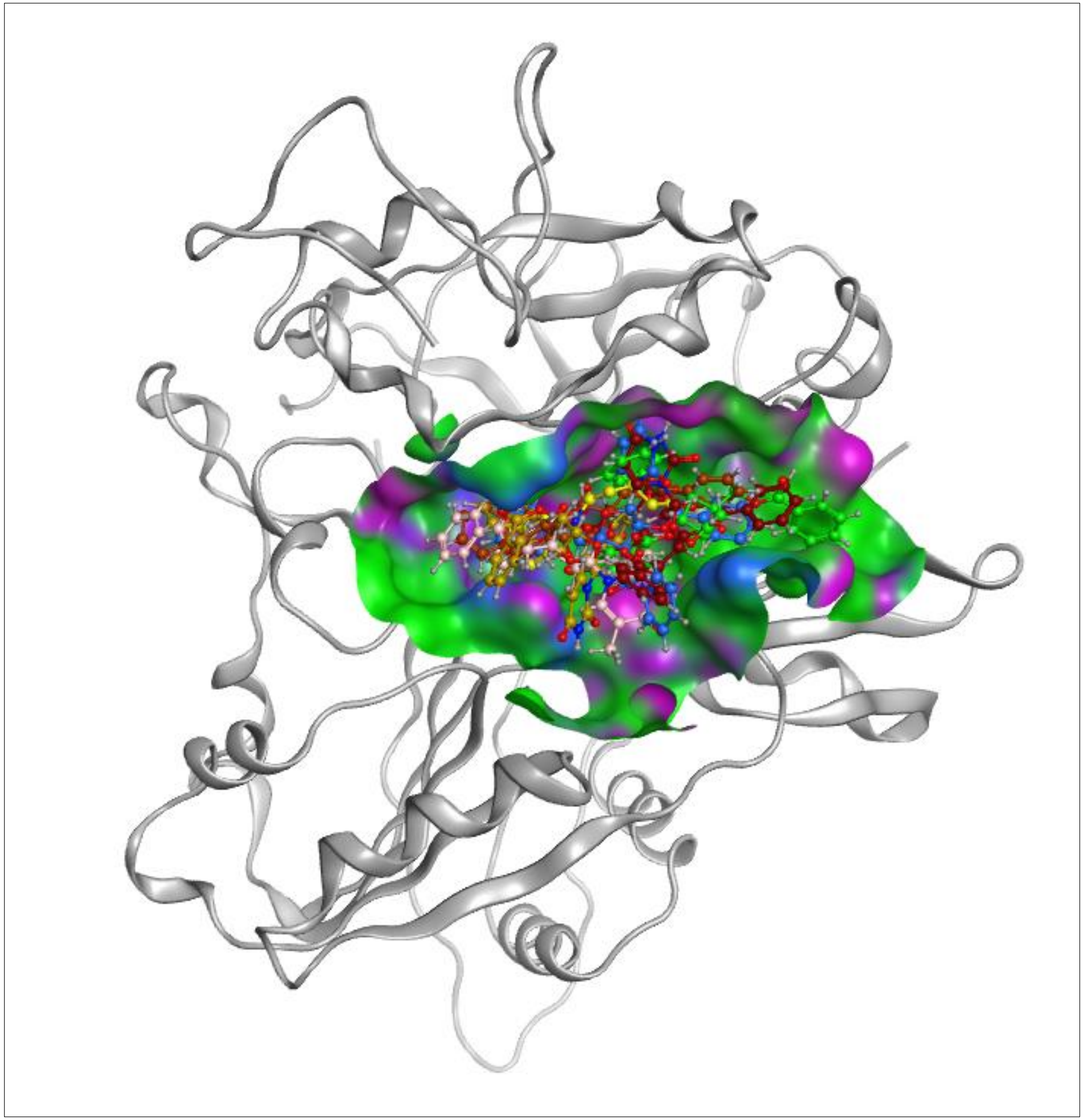

Fig. S28. Superposition of **2D-2L**, **3D-3L**, **4D-4′D** and inhibitor XZ578 in a crystalline complex with Tdp1 (PDB ID: 6n19).

Fig. S29. Molecular docking models for allosteric Tdp1 inhibitors (ribbon model)

***D*-nucleoside 3D**	***L*-nucleoside 3L**
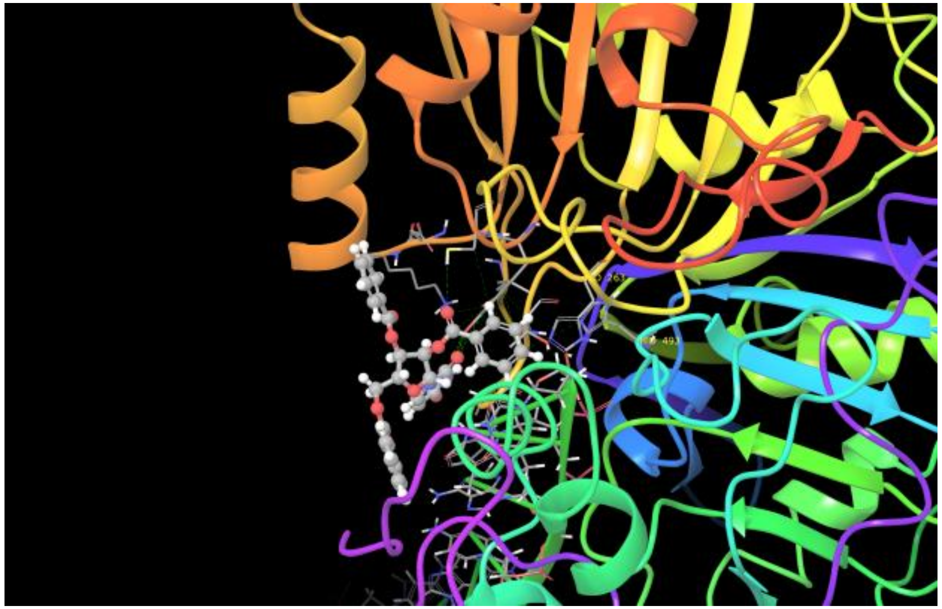	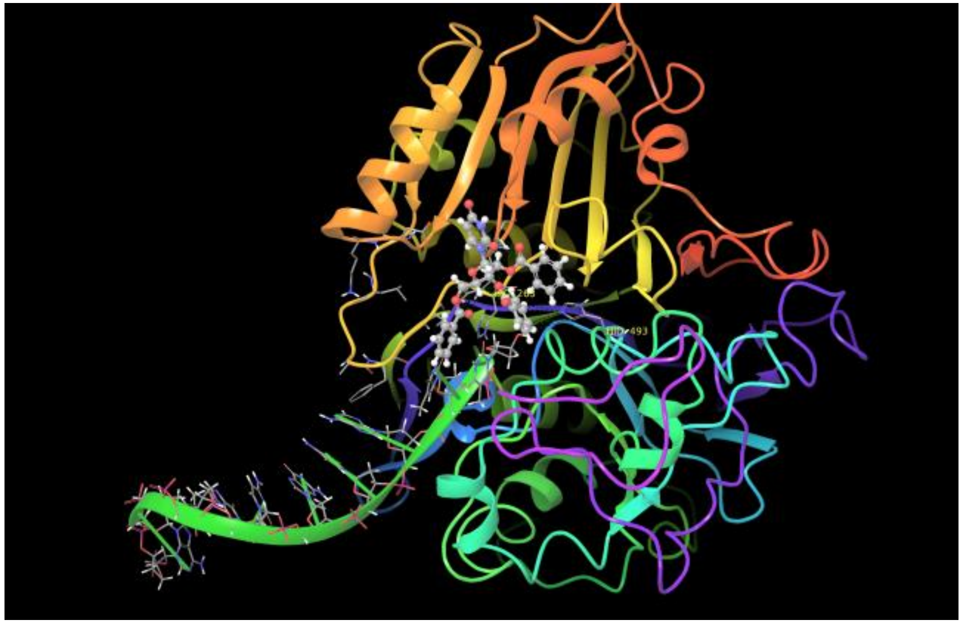

Fig. S30. Molecular docking models for competitive Tdp1 inhibitors (ribbon model)	
***5′R*-nucleoside 7R**	***5′S*-nucleoside 7S**
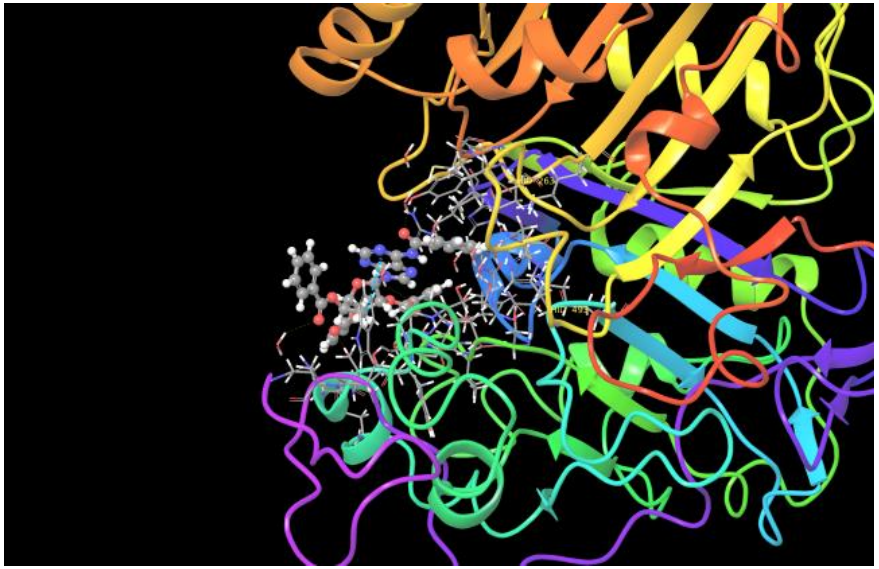	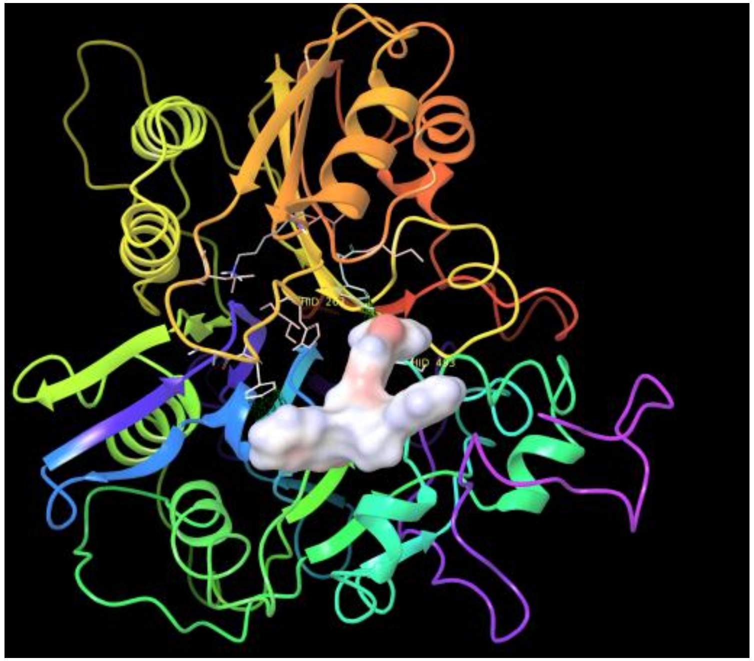

Fig. S31. Molecular docking models for Tdp1 inhibitors 4D and 4L (electrostatic interactions)

4D

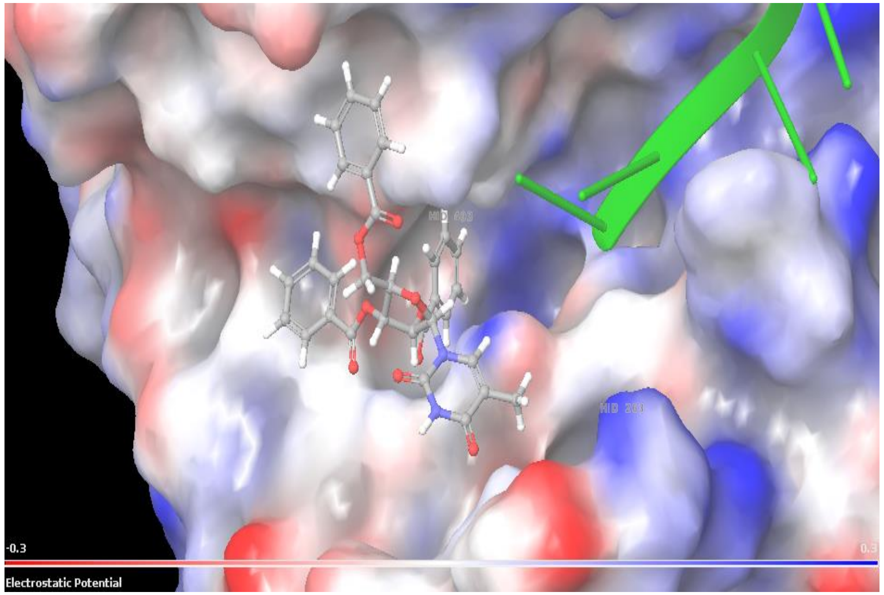

4L

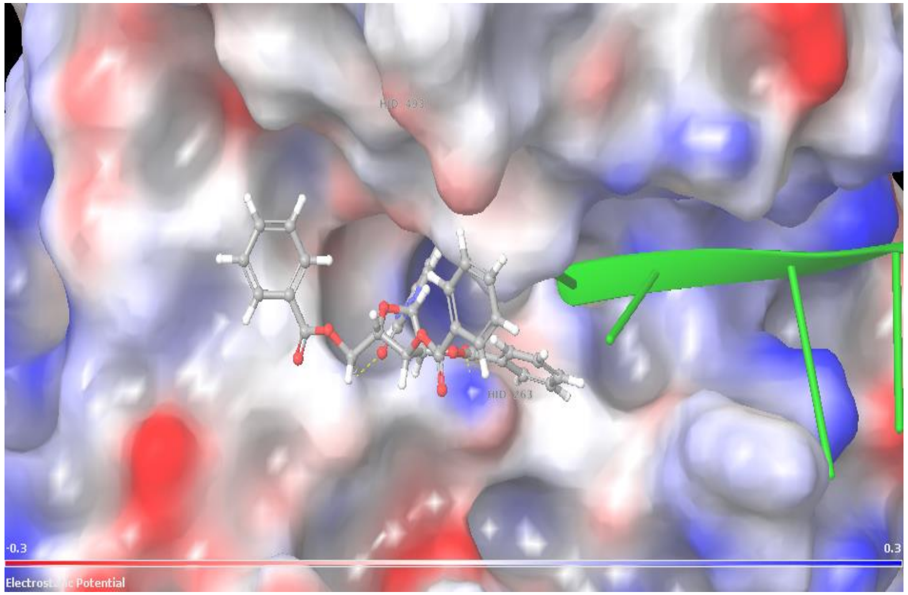

Fig. S32. 2D ligand interactions for Tdp1 inhibitors 4D and 4L

4D	4L
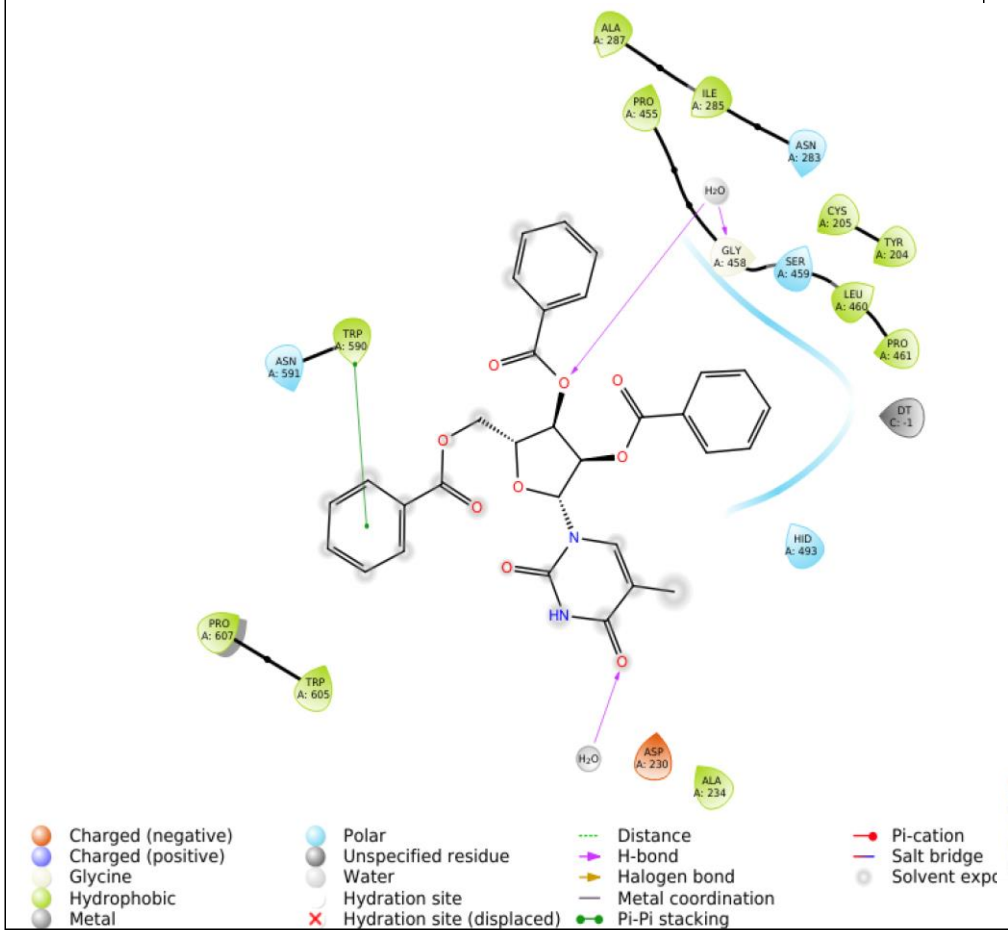	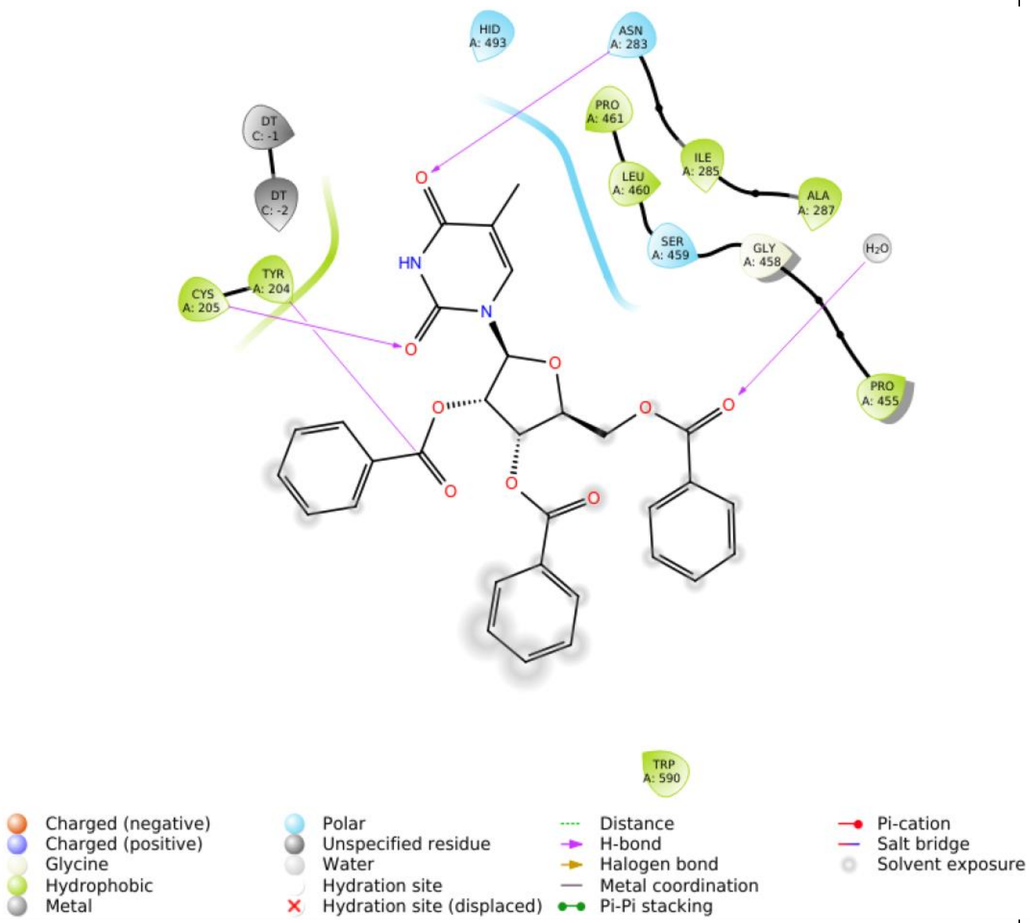

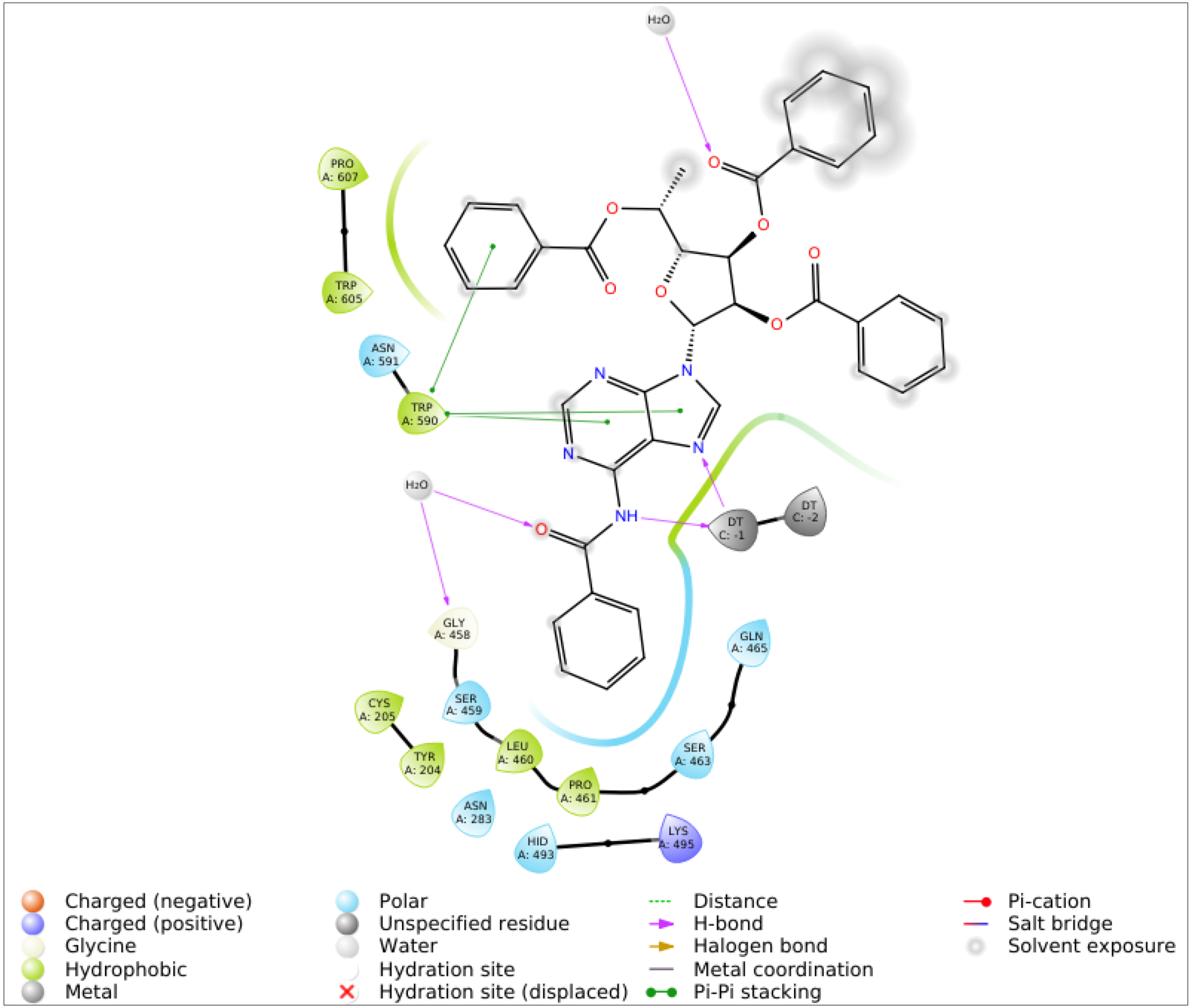

*Fig. S33. 2D ligand interactions for Tdp1 inhibitor **7R** in complex with DNA.*

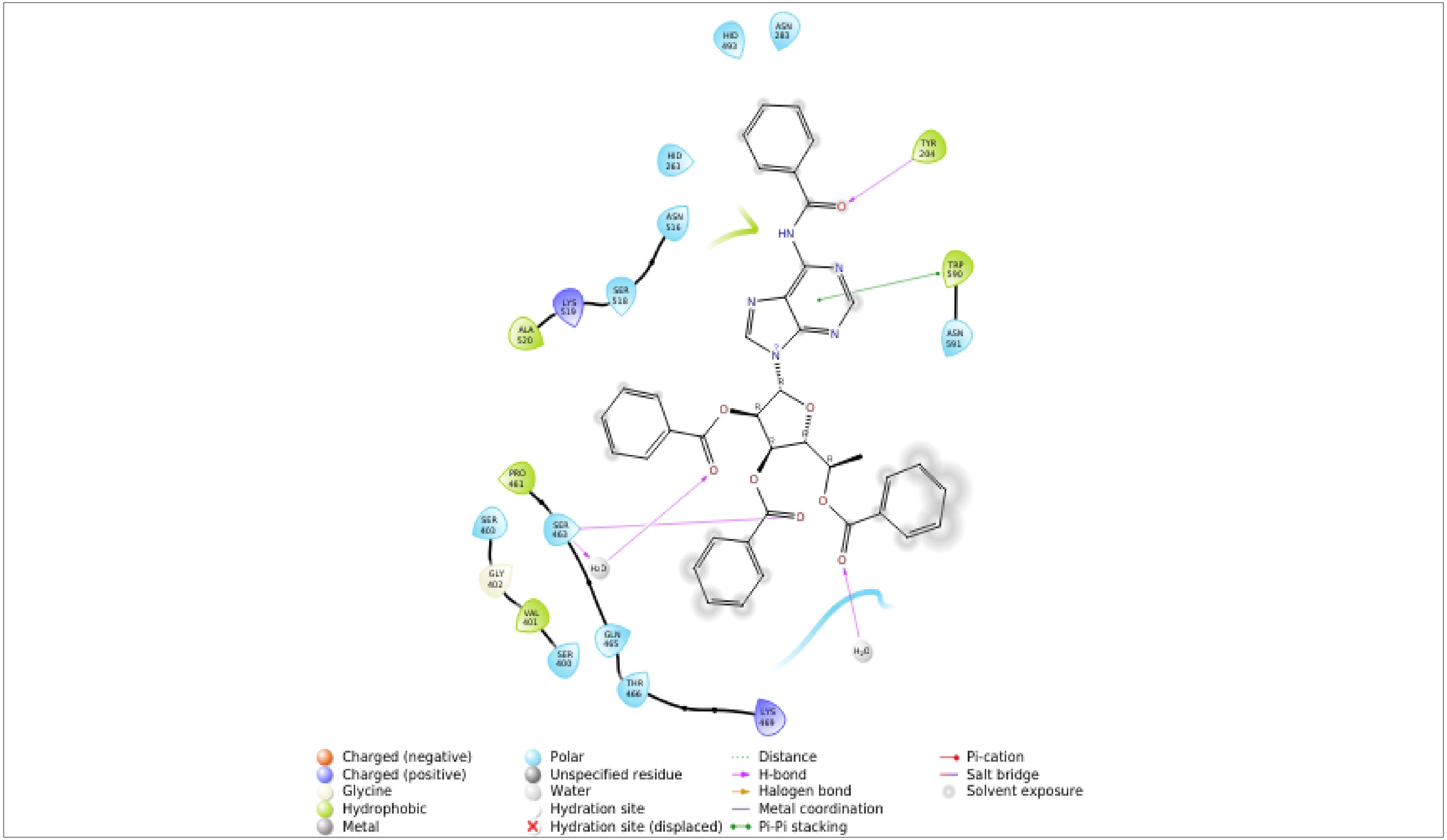

Fig. S34. 2D ligand interactions for Tdp1 inhibitor ***7R*** *in apo-form of Tdp1.*

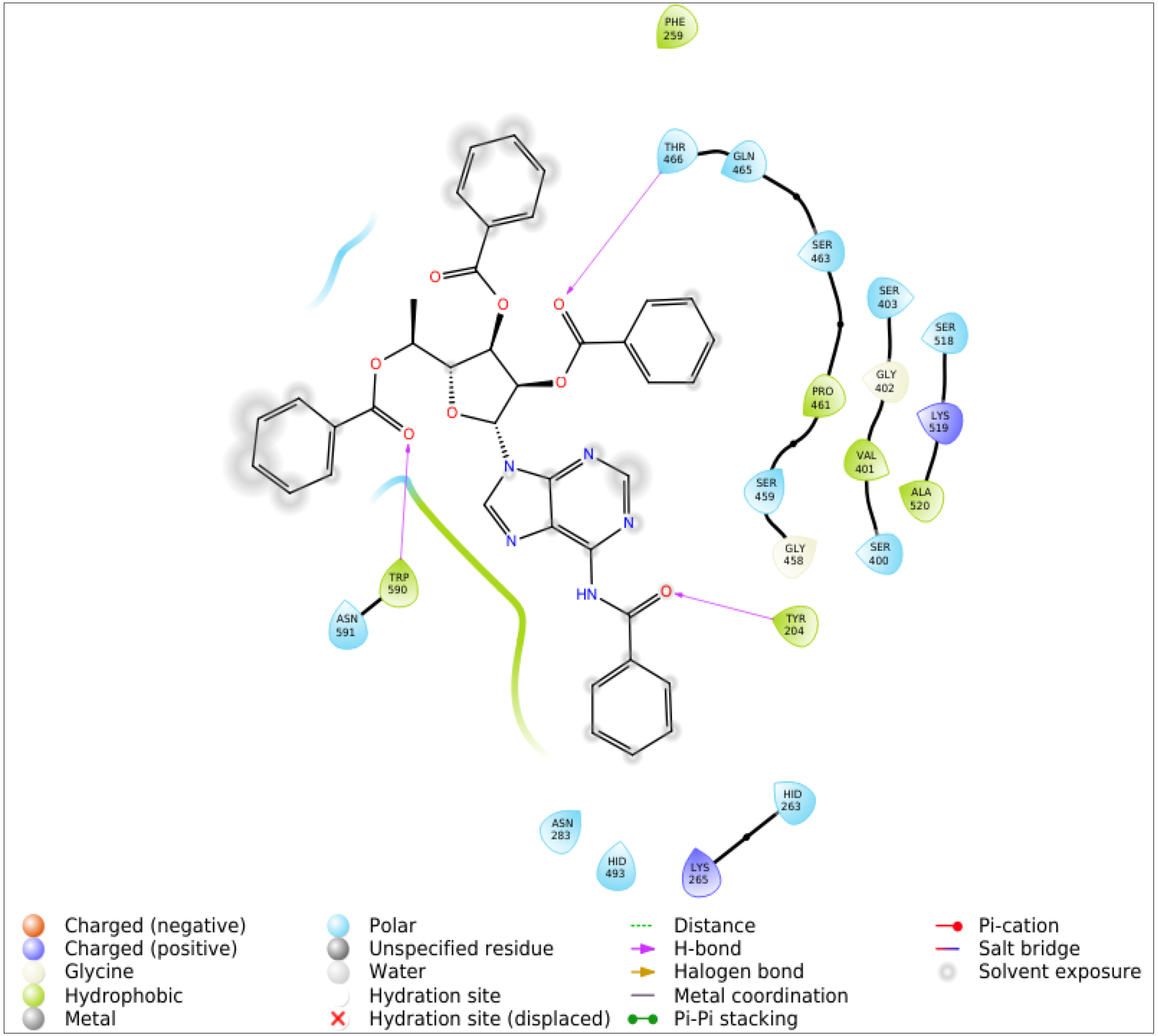

*Fig. S35. 2D ligand interactions for Tdp1 inhibitor **7S** in apo-form of Tdp1.*

Table S1. Binding energy (docking_score) and binding propensity (glide emodel) evaluation for 24 optimal positions of conformers of **3D**/**3L** in complex with Tdp1-DNA (sorting by docking score).

**glide-dock_XP_complexDNA_473**	docking score	glide emodel		**glide-dock_XP_complexDNA_567**	docking score	glide emodel
**3D**	-5,996	-60,547		**3L**	-6,161	-62,135
**3D**	-5,888	-57,226		**3L**	-5,805	-74,622
**3D**	-5,735	-61,825		**3L**	-5,719	-65,002
**3D**	-5,608	-60,695		**3L**	-5,706	-61,796
**3D**	-5,456	-59,199		**3L**	-5,683	-62,620
**3D**	-5,355	-53,776		**3L**	-5,530	-60,325
**3D**	-5,289	-57,218		**3L**	-5,482	-59,488
**3D**	-5,260	-53,919		**3L**	-5,278	-63,713
**3D**	-5,255	-56,348		**3L**	-5,274	-58,799
**3D**	-5,244	-54,557		**3L**	-5,256	-65,591
**3D**	-5,241	-57,772		**3L**	-5,181	-57,752
**3D**	-5,235	-58,413		**3L**	-5,152	-68,020
**3D**	-5,233	-61,334		**3L**	-5,149	-63,238
**3D**	-5,176	-59,070		**3L**	-5,122	-62,060
**3D**	-5,168	-57,578		**3L**	-5,080	-71,471
**3D**	-5,152	-55,796		**3L**	-5,060	-59,432
**3D**	-5,022	-55,698		**3L**	-5,046	-70,511
**3D**	-5,016	-63,838		**3L**	-4,973	-60,246
**3D**	-4,994	-63,699		**3L**	-4,962	-70,644
**3D**	-4,983	-52,912		**3L**	-4,904	-58,414
**3D**	-4,876	-53,366		**3L**	-4,840	-61,720
**3D**	-4,735	-60,328		**3L**	-4,737	-57,612
**3D**	-4,707	-56,847		**3L**	-4,732	-60,310
**3D**	-4,557	-52,519		**3L**	-4,651	-57,560

Table S2. Binding energy evaluation for 24 optimal positions of conformers of **4D**/**4L** in complex with Tdp1-DNA (sorting by docking score).

**glide-dock_XP_complexDNA_566**	docking score	glide emodel		**glide-dock_XP_complexDNA_568**	docking score	glide emodel
**4D**	-5,109	-62,013		**4L**	-5,927	-63,121
**4D**	-4,949	-58,522		**4L**	-5,895	-65,212
**4D**	-4,927	-66,784		**4L**	-5,867	-64,146
**4D**	-4,925	-69,370		**4L**	-5,814	-63,213
**4D**	-4,902	-67,312		**4L**	-5,612	-62,863
**4D**	-4,893	-62,919		**4L**	-5,608	-63,382
**4D**	-4,893	-62,757		**4L**	-5,588	-52,675
**4D**	-4,891	-66,559		**4L**	-5,546	-52,486
**4D**	-4,890	-66,738		**4L**	-5,453	-65,554
**4D**	-4,886	-67,374		**4L**	-5,400	-64,936
**4D**	-4,871	-67,028		**4L**	-5,278	-72,999
**4D**	-4,856	-70,922		**4L**	-5,239	-61,890
**4D**	-4,848	-62,674		**4L**	-5,221	-64,404
**4D**	-4,803	-67,241		**4L**	-5,215	-70,683
**4D**	-4,704	-56,469		**4L**	-5,203	-57,247
**4D**	-4,657	-61,535		**4L**	-5,196	-59,617
**4D**	-4,654	-56,733		**4L**	-5,172	-58,749
**4D**	-4,653	-68,088		**4L**	-5,136	-61,498
**4D**	-4,615	-61,300		**4L**	-5,127	-66,866
**4D**	-4,376	-65,019		**4L**	-5,067	-64,909
**4D**	-4,375	-61,845		**4L**	-5,066	-61,398
**4D**	-4,094	-62,952		**4L**	-5,045	-63,013
**4D**	-3,968	-65,835		**4L**	-5,037	-60,443
**4D**	-3,341	-56,740		**4L**	-4,974	-61,710

Table S3. Binding energy evaluation for 24 optimal positions of conformers of **7R/7S** in complex with apo-form of Tdp1 (sorting by docking score).

**glide-dock_XP_6mj9_str15**	docking score	glide emodel		**glide-dock_XP_6mj9_str604**	docking score	glide emodel
**7R**	-2,843	-81,669		**7S**	-5,002	-83,946
**7R**	-2,649	-83,942		**7S**	-4,450	-74,563
**7R**	-2,360	-80,977		**7S**	-4,213	-79,799
**7R**	-2,257	-86,203		**7S**	-4,193	-72,589
**7R**	-2,205	-82,141		**7S**	-3,541	-70,527
**7R**	-1,815	-84,406		**7S**	-3,240	-70,172
**7R**	-1,716	-89,722		**7S**	-3,169	-76,743
**7R**	-1,510	-80,861		**7S**	-3,123	-70,285
**7R**	-1,311	-79,576		**7S**	-2,962	-83,496
**7R**	-1,233	-82,787		**7S**	-2,903	-83,395
**7R**	-1,213	-82,738		**7S**	-2,749	-84,779
**7R**	-1,055	-86,647		**7S**	-2,601	-81,931
**7R**	-1,000	-82,320		**7S**	-2,311	-106,485
**7R**	-0,972	-76,528		**7S**	-2,253	-85,450
**7R**	-0,946	-81,438		**7S**	-1,915	-100,871
**7R**	-0,811	-80,904		**7S**	-1,666	-82,790
**7R**	-0,760	-86,234		**7S**	-1,556	-85,636
**7R**	-0,695	-76,946		**7S**	-1,431	-74,006
**7R**	-0,667	-90,880		**7S**	-1,412	-88,241
**7R**	-0,665	-85,433		**7S**	-1,251	-79,883
**7R**	-0,648	-81,549		**7S**	-1,115	-75,677
**7R**	-0,624	-81,042		**7S**	-1,049	-83,387
**7R**	-0,623	-86,850		**7S**	-1,023	-93,129
**7R**	-0,586	-84,029		**7S**	-1,021	-82,110

Table S4. Binding energy evaluation for 24 optimal positions of conformers of **7R** in complex with Tdp1-DNA (sorting by docking score).

**glide-dock_XP_complexDNA_15**	docking score	glide emodel
**7R**	-8,547	-79,079
**7R**	-7,915	-75,840
**7R**	-7,620	-81,147
**7R**	-7,503	-77,661
**7R**	-7,370	-74,606
**7R**	-7,308	-70,915
**7R**	-7,177	-80,157
**7R**	-6,980	-81,454
**7R**	-6,733	-70,615
**7R**	-6,704	-83,243
**7R**	-6,592	-80,873
**7R**	-6,506	-73,331
**7R**	-6,270	-80,484
**7R**	-6,233	-82,584
**7R**	-6,187	-77,861
**7R**	-6,118	-75,451
**7R**	-6,026	-80,643
**7R**	-5,956	-71,321
**7R**	-5,932	-78,085
**7R**	-5,867	-83,397
**7R**	-5,780	-77,141
**7R**	-5,641	-76,862
**7R**	-5,609	-74,698
**7R**	-5,530	-82,715
**7R**	-5,333	-82,603
**7R**	-5,300	-84,674

3D (uncompetitive)

A

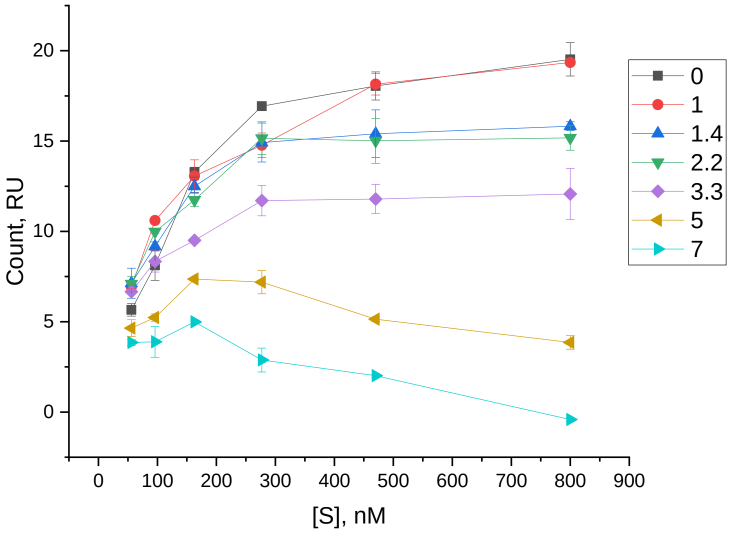

B

		Value	Error
0	Vmax	27	3
0	Km	171	39
1	Vmax	22,0	0,3
1	Km	108	6
1.4	Vmax	17,6	0,5
1.4	Km	81	9
2.2	Vmax	17,1	0,8
2.2	Km	69	8
3.3	Vmax	12,3	0,2
3.3	Km	48	3
5	Vmax	7	1
5	Km	0	32
7	Vmax	2	1
7	Km	0	82

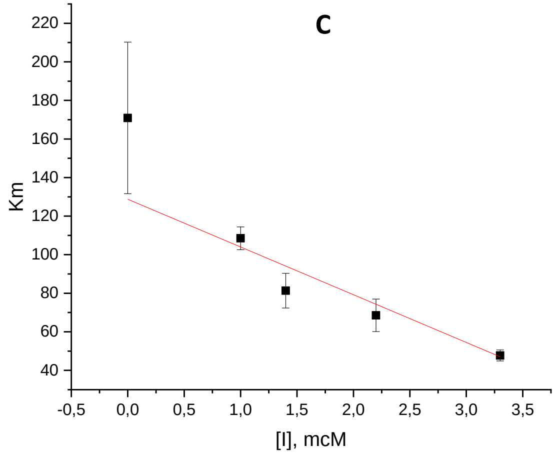

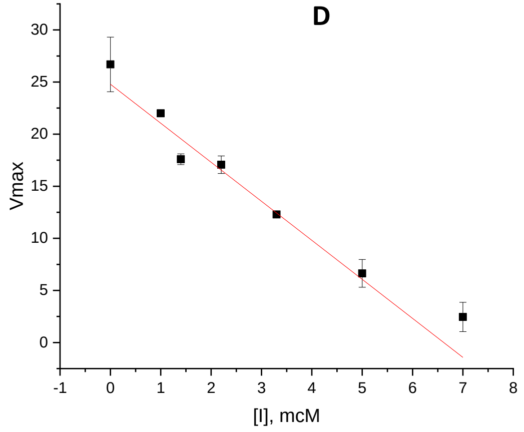

Fig. S36. The type of inhibition of compound **3D**. A. Curves of the fluorescence intensity dependence of the intensity on the substrate concentration (S) in the presence of different concentrations of the inhibitor. **B.** Vmax and Km values at different inhibitor concentrations. **C.** Graph of Km versus inhibitor concentration. **D.** Graph of Vmax versus inhibitor concentration.

3L (uncompetitive)

A

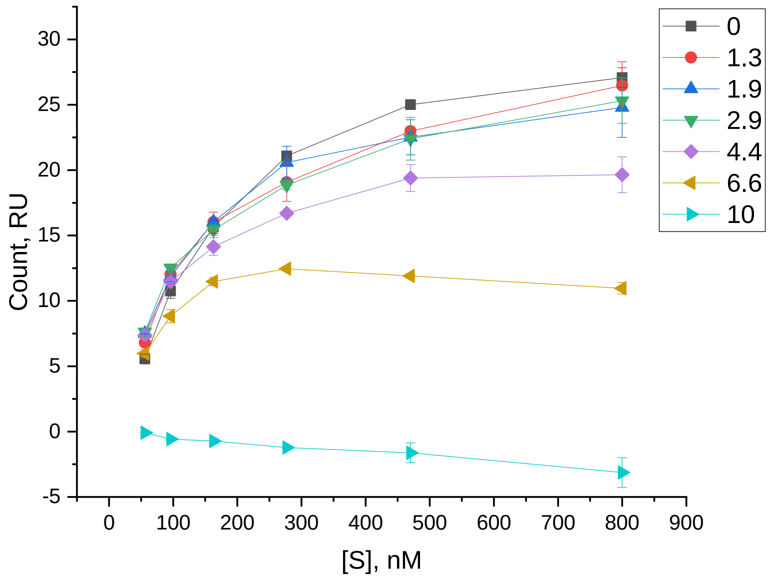

B

		Value	Error
0	Vmax	37	3
0	Km	221	44
1.3	Vmax	34	5
1.3	Km	176	45
1.9	Vmax	39	2
1.9	Km	233	14
2.9	Vmax	28	2
2.9	Km	129	19
4.4	Vmax	23	1
4.4	Km	106	11
6.6	Vmax	14	1
6.6	Km	43	23
10	Vmax	0	2
10	Km	463	0

C

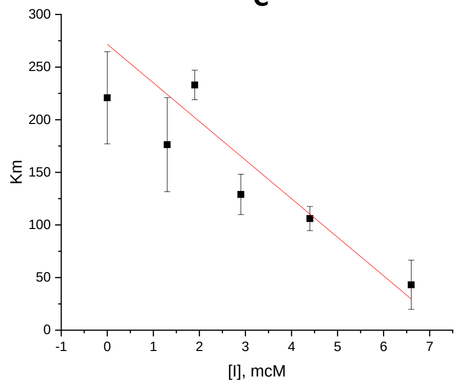

D

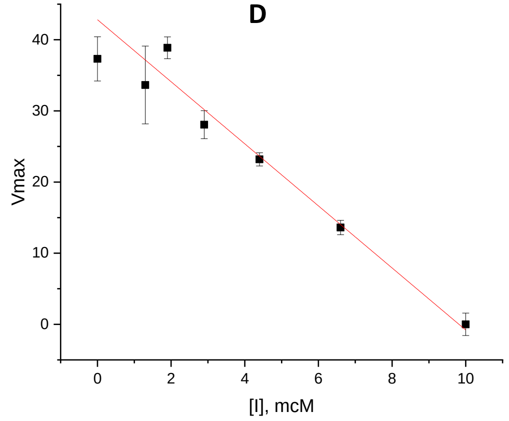

Fig. S37. The type of inhibition of compound **3L**. A. Curves of the fluorescence intensity dependence of the intensity on the substrate concentration (S) in the presence of different concentrations of the inhibitor. **B.** Vmax and Km values at different inhibitor concentrations. **C.** Graph of Km versus inhibitor concentration. **D.** Graph of Vmax versus inhibitor concentration.

7S (competitive)

A

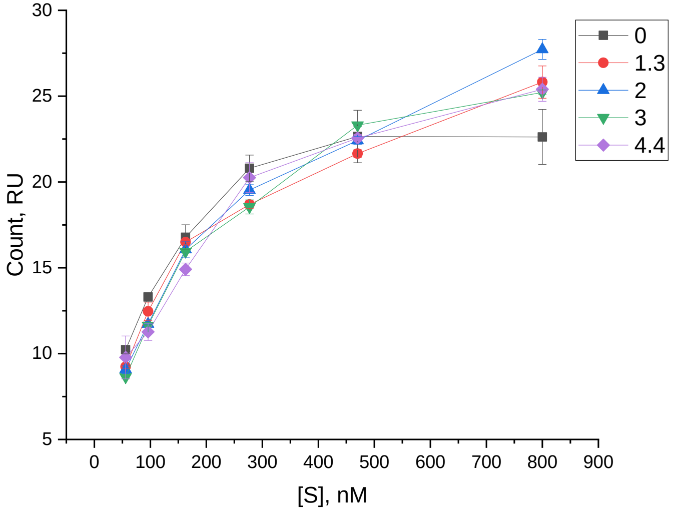

B

		Value	Error
0	Vmax	26	1
0	Km	93	9
1.3	Vmax	26,5	0,7
1.3	Km	104	6
2	Vmax	30	1
2	Km	150	11
3	Vmax	30,7	0,6
3	Km	154	7
4.4	Vmax	30,4	0,8
4.4	Km	164	14

C

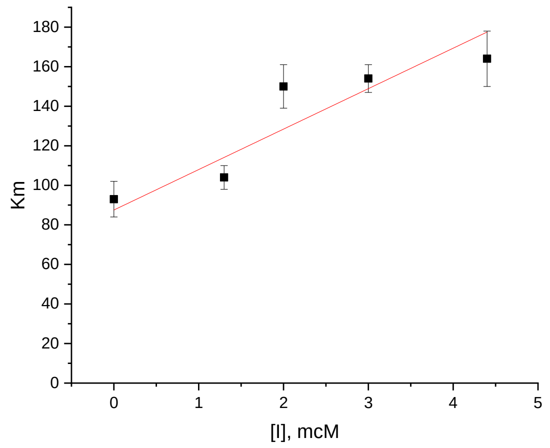

D

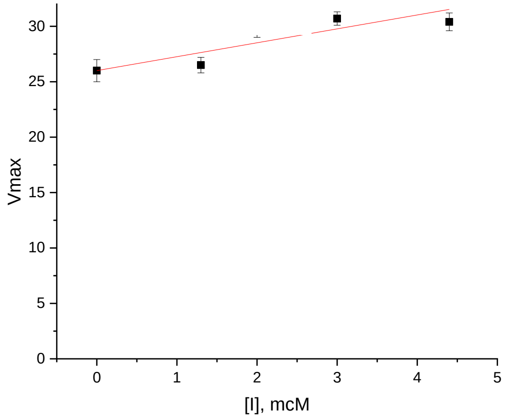

Fig. S38. The type of inhibition of compound **7S**. A. Curves of the fluorescence intensity dependence of the intensity on the substrate concentration (S) in the presence of different concentrations of the inhibitor. **B.** Vmax and Km values at different inhibitor concentrations. **C.** Graph of Km versus inhibitor concentration. **D.** Graph of Vmax versus inhibitor concentration.

7R (mixed)

A

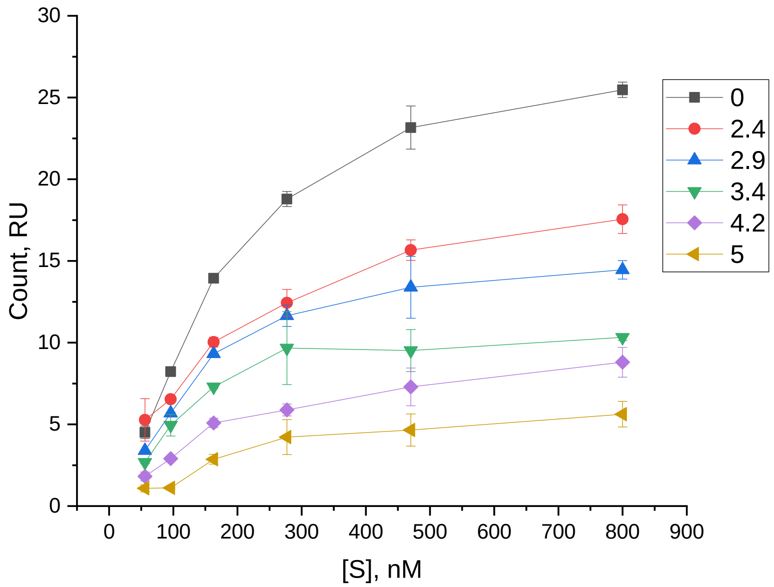

B

		Value	Standard Error
0	Vmax	35,5	2,8
0	Km	274,4	51,3
2,4	Vmax	22,1	0,8
2,4	Km	204,6	18,8
2,9	Vmax	18,5	1,3
2,9	Km	187,7	35,0
3,4	Vmax	12,7	1,2
3,4	Km	138,8	39,9
4,2	Vmax	10,1	0,8
4,2	Km	200,2	39,5
5	Vmax	8,2	1,3
5	Km	338,8	45,2

C

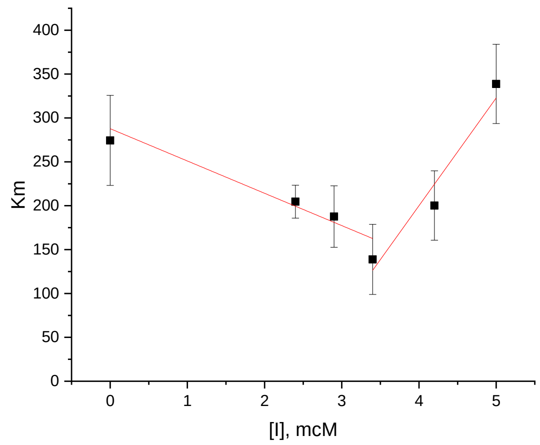

Fig. S39. The type of inhibition of compound **7R**. A. Curves of the fluorescence intensity dependence of the intensity on the substrate concentration (S) in the presence of different concentrations of the inhibitor. **B.** Vmax and Km values at different inhibitor concentrations. **C.** Graph of Km versus inhibitor concentration. **D.** Graph of Vmax versus inhibitor concentration.

D

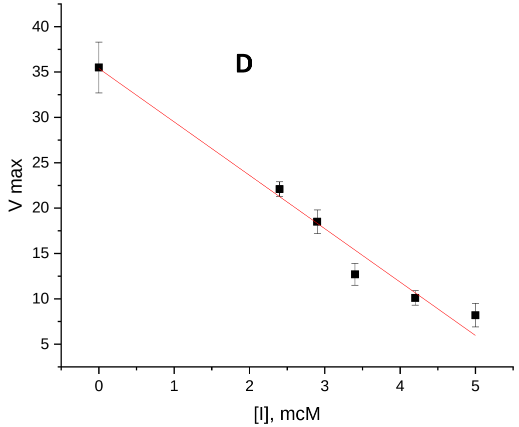

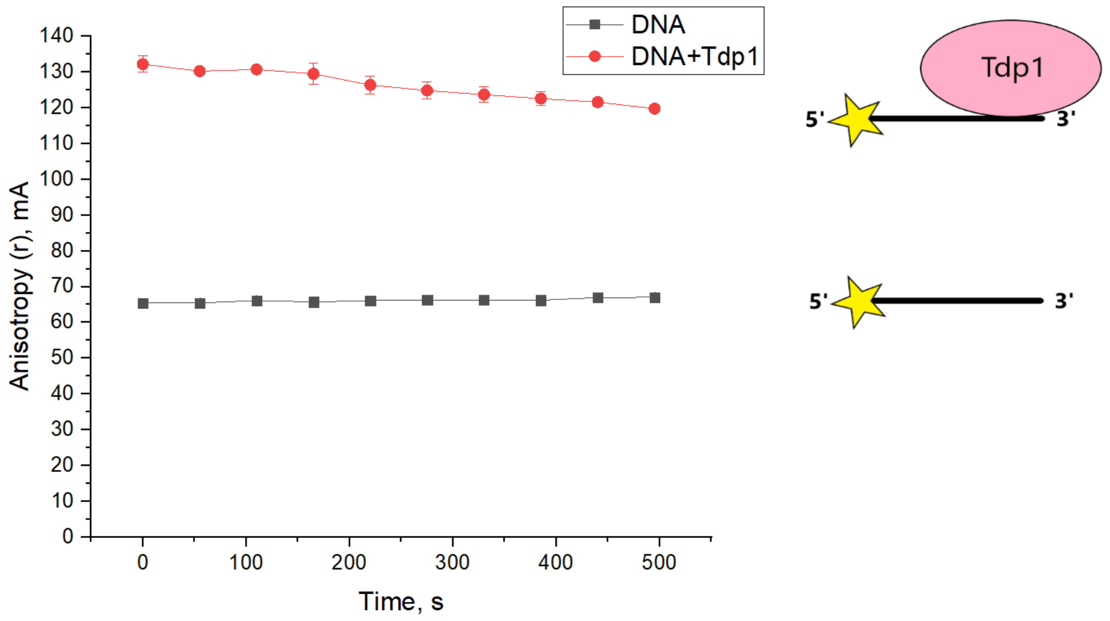

Fig. S40. Anisotropy of biosensor fluorescence in the presence and absence of Tdp1.
